# A dynamic thermoregulatory material inspired by squid skin

**DOI:** 10.1038/s41467-019-09589-w

**Published:** 2019-04-29

**Authors:** Erica M. Leung, Melvin Colorado Escobar, George T. Stiubianu, Steven R. Jim, Alexandra L. Vyatskikh, Zhijing Feng, Nicholas Garner, Priyam Patel, Kyle L. Naughton, Maurizio Follador, Emil Karshalev, Matthew D. Trexler, Alon A. Gorodetsky

**Affiliations:** 1 Department of Chemical Engineering and Materials Science, University of California, Irvine, Irvine, CA 92697 USA; 2 Department of Chemistry, University of California, Irvine, Irvine, CA 92697 USA; 3 Under Armour, Inc., Baltimore, MD 21230 USA; 4 Department of Physics, University of California, Irvine, Irvine, CA 92697 USA

**Keywords:** Energy science and technology, Materials for optics

## Abstract

Effective thermal management is critical for the operation of many modern technologies, such as electronic circuits, smart clothing, and building environment control systems. By leveraging the static infrared-reflecting design of the space blanket and drawing inspiration from the dynamic color-changing ability of squid skin, we have developed a composite material with tunable thermoregulatory properties. Our material demonstrates an on/off switching ratio of ~25 for the transmittance, regulates a heat flux of ~36 W/m^2^ with an estimated mechanical power input of ~3 W/m^2^, and features a dynamic environmental setpoint temperature window of ~8 °C. Moreover, the composite can manage one fourth of the metabolic heat flux expected for a sedentary individual and can also modulate localized changes in a wearer’s body temperature by nearly 10-fold. Due to such functionality and associated figures of merit, our material may substantially reduce building energy consumption upon widespread deployment and adoption.

## Abstract (web summary)

Thermoregulatory platforms that combine the advantages of passive and active thermal management systems have remained elusive. Here, the authors draw inspiration from the static infrared-reflecting space blanket and dynamic color-changing squid skin to develop a composite material that addresses this challenge.

## Introduction

Effective management of heat transfer enables the operation of many ubiquitous modern technologies, including electronic circuits^1^, aircraft and spacecraft components^2^, clinical warming devices^3^, power generation platforms^4^, shipping and packaging containers^5^, specialty textiles and garments^6^, and building environment control systems^7^. Within this context, building operation accounts for ~40% of global energy consumption (with heating and cooling alone requiring ~36% of this amount)^7,8^, and as such, the development of novel personal (localized or wearable) thermoregulatory platforms represents an exciting opportunity and can dramatically diminish energy use worldwide^9^. To date, the great variety and abundance of indoor thermal management systems have been classified as either “passive” or “active,” depending on the underlying mode of operation^10–13^. Specifically, representative passive heating technologies, such as insulation, textiles, and reflective coatings, employ materials with low thermal conductivities and/or high infrared reflectances, and thus regulate temperature by blocking heat transfer (Supplementary Table 1). These systems are low cost, energy efficient, and simple to implement but are also static and unresponsive to changing conditions^10–13^. In contrast, representative active heating technologies, such as electrothermal devices and heating/ventilation/air conditioning platforms, regulate temperature by driving the flow of heat through the external input of electrical and/or mechanical energy (Supplementary Table 1). These systems are dynamic and readily allow for user control but are also relatively expensive, energy inefficient, and complex to install^10–13^. Consequently, it is highly desirable to develop an “ideal” thermal management platform that merges the advantages of passive systems (i.e., low cost, straightforward implementation, and energy efficiency) with the on-demand dynamic control capabilities of active systems.

**Fig. 1:**
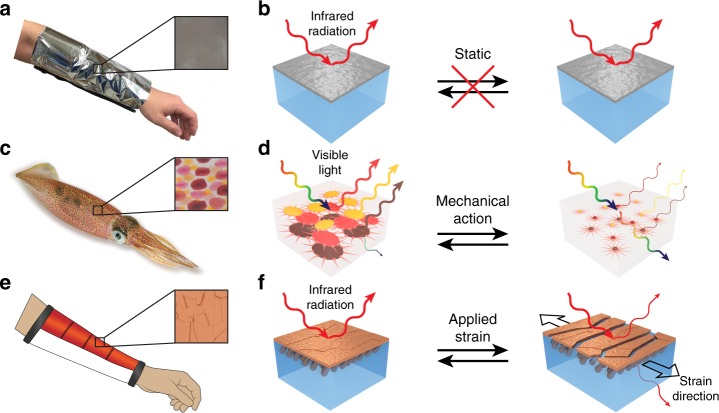
Bioinspired design of the thermoregulatory composite material. **a** Camera image of a space blanket on a human arm. The space blanket’s outer surface is a continuous metal film (inset). **b** Schematics of the space blanket, which consists of a plastic (e.g., polyethylene terephthalate) sheet overlaid with a continuous metal (e.g., aluminum) layer. For this static system, an external stimulus cannot modulate the reflection and transmission of infrared radiation (left and right). **c** Camera image of a squid. The skin contains red, yellow, or brown chromatophore organs as illustrated by the representative inset picture. **d** Schematics of a layer found in squid skin, which consists of arrayed chromatophore organs embedded within a visibly-transparent dermal matrix. For this dynamic system, the mechanical action of muscle cells switches the chromatophores between expanded plate-like (left) and contracted point-like (right) states and thus modulates the reflection and transmission of specific wavelengths of visible light. **e** Schematic of the composite material on a human arm in a wearable (sleeve) configuration. The composite’s outer surface is a fractured multi-domain metal (e.g., copper) coating (inset). **f** Schematics of the composite material, which consists of an infrared-transparent polymer matrix overlaid with infrared-reflecting nanostructure-anchored metal domains. For this dynamic system, an applied strain switches the metal domain arrangement from densely (left) to sparsely (right) packed and thus modulates the reflection and transmission of infrared radiation (e.g., heat). Panel **c** is copyright (c) 2019 pngimg.com and made available under an Attribution-NonCommercial 4.0 International Public License. Panel **c** inset is copyright (c) 2012 Backyard Brains and made available under an Attribution-ShareAlike 3.0 United States Public License

Among passive thermal management systems, the “space blanket,” which was introduced by NASA in the 1960s to mitigate temperature fluctuations in space, represents arguably one of the most famous and impactful wearable technologies reported to date (Fig. 1a)^14^. In its standard configuration, the space blanket consists of a plastic sheet (e.g., polyethylene terephthalate) overlaid with a thin continuous layer of metal (e.g., aluminum) (Fig. 1a, inset and Fig. 1b, left)—an architecture that is highly effective at reflecting infrared radiation (e.g., heat)^14^. Various incarnations of the space blanket have found applications in packaging, emergency portable shelters, clinical warming devices, and protective or performance apparel^3,5,6,14–17^. However, the space blanket’s application scope has been limited by its static thermal properties, which cannot be reconfigured on demand or adjusted in any way (Fig. 1b). Indeed, as a technology, the space blanket has remained fundamentally unchanged over the past 50 years^14^.

Among active thermal management systems, recent state-of-the-art efforts have focused on the development of components with switching capabilities^18–20^. These devices consist of stimuli-responsive materials, e.g., metal oxide layers or conducting polymer films, which are placed within a gap or junction (Supplementary Table 2)^18–20^. In such configurations, the heat transferred through (by) the material is dynamically modulated (switched) with an external non-thermal input, e.g., a mechanical pressure or an applied voltage (Supplementary Table 2)^18–20^. Thermal switches (and analogous components) have been touted as promising for applications like refrigeration and energy storage but are rarely mentioned within the context of personal thermal management^18–20^. Indeed, for effective implementation in wearable thermoregulatory systems, thermal switches must satisfy numerous demanding criteria, including ease of manufacturing over large areas, a straightforward actuation method, reversibility and tunability without hysteresis, stability to repeated cycling, a soft and stretchable form factor, rapid response time, a high on/off ratio, and a low operating temperature (Supplementary Table 2). Thus far, no single thermal switching technology has been shown to simultaneously possess all of these characteristics.

The fascinating dynamic color-changing skin of coleoid cephalopods (squid, octopuses, and cuttlefish) represents a judicious source of inspiration for next-generation adaptive thermal management systems (Fig. 1c)^21–23^. As one example, squid skin consists of multiple layers, one of which contains embedded red, yellow, and brown chromatophore organs (Fig. 1c, inset and Fig. 1d, left)^24–27^. The organs, which are arranged in an arrayed pattern, consist of a central pigment cell ringed by innervated muscle cells^24–27^. This highly evolved natural architecture allows for the pigment cells to be dynamically switched by the muscle cells between contracted point-like and expanded plate-like states, thereby modulating the local coloration and changing the transmission of light through the skin (Fig. 1d)^24–27^. Accordingly, the unique structure and function of cephalopod skin has motivated the engineering of various unconventional color- and appearance-changing technologies, including biomimetic soft active surfaces^28^, optoelectronic displays^29^, electromechanochemically responsive elastomers^30^, stretchable electroluminescent materials^31^, and adaptive infrared camouflage^32^. In this regard, natural squid skin and its constituent components exhibit nearly all of the capabilities required for wearable thermal switches (Supplementary Table 2), making them promising models for novel thermoregulatory platforms.

Herein, we draw inspiration from the static infrared-reflecting space blanket and active color-changing squid skin to design and develop a tunable thermoregulatory material. First, we fabricate a bioinspired composite material via common manufacturing techniques. Next, we characterize the composite’s mechanical characteristics and ability to manage infrared radiation. Subsequently, we model and evaluate our material’s stimuli-responsive thermal management properties. Last, we demonstrate its ability to regulate human body temperature in a wearable configuration. Overall, the composite material may shift energy consumption paradigms for a variety of modern technologies.

## Results

### Bioinspired design

We envisioned a personal thermal management technology, which would leverage the static infrared-reflecting configuration of the space blanket and draw inspiration from the dynamic color-changing mechanisms intrinsic to squid skin (Fig. 1e). We thus designed a composite material comprised of (1) a soft and stretchable infrared-transparent polymer matrix and (2) an overlaid array of infrared-reflecting metal domains stably anchored within the matrix via columnar nanostructures (Fig. 1e, inset and Fig. 1f, left). In our design, the polymer matrix emulates the chromatophore-containing transparent dermal layer of squid, while the metal domains emulate the embedded chromatophore organs themselves. Prior to any mechanical actuation, the nanostructure-anchored, infrared-reflecting metal domains are densely packed and completely cover the underlying matrix (Fig. 1f, left), in similar fashion to how expanded plate-like chromatophores are overlapped in squid skin (Fig. 1d, left). The composite material thus reflects nearly all incident infrared radiation (Fig. 1f, left), in analogy to the arrays of overlapped chromatophores reflecting visible light of specific wavelengths (Fig. 1d, left). However, upon mechanical actuation, the anchored metal domains become spread apart and uncover portions of the stretched underlying polymer matrix (Fig. 1f, right), in similar fashion to how contracted point-like chromatophores are separated in squid skin (Fig. 1d, right). The composite material thus transmits a significant fraction of the incident infrared radiation (Fig. 1f, right), in analogy to arrays of contracted chromatophores transmitting relatively more visible light (Fig. 1d, right). In essence, mechanical actuation (stretching) reversibly changes the surface microstructure of our composite material and thus dynamically alters its ability to transmit and reflect infrared radiation (e.g., heat). The general design simultaneously encompasses the desirable technical advantages of the space blanket (i.e., straightforward low-cost manufacturability and outstanding energy efficiency) and the unique natural characteristics of squid skin (i.e., a favorable form factor and on-demand controllability).

### Composite fabrication

**Fig. 2:**
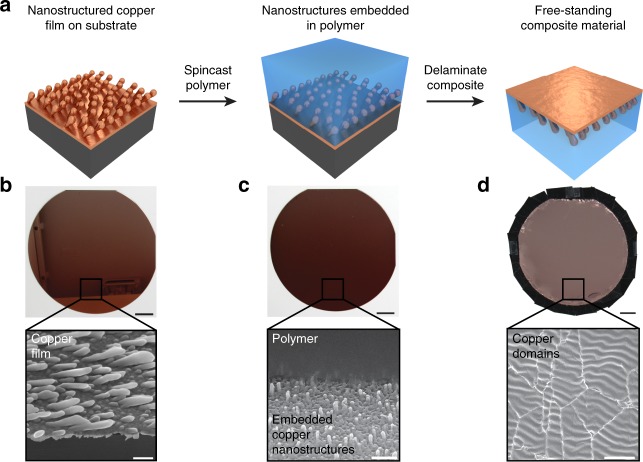
Preparation of the thermoregulatory composite material. **a** Schematic of the general fabrication procedure for the composite material. The steps consist of the electron-beam deposition of a nanostructured copper film onto a support substrate (left), the spincasting of a styrene–ethylene–butylene–styrene block copolymer directly onto this film (middle), and the delamination of the resulting composite from the substrate (right). **b** Digital camera image of a substrate-bound nanostructured copper film (top). The scale bar is 2 cm. A representative top–down scanning electron microscopy (SEM) image demonstrates that the film consists of arrayed tilted nanoscale columns that emerge from an underlying continuous copper coating (inset). The scale bar is 200 nm. **c** Digital camera image of a substrate-bound composite material (top). The scale bar is 2 cm. A representative cross-sectional SEM image demonstrates that the tilted copper nanostructures are anchored within the polymer (inset). The scale bar is 500 nm. **d** Digital camera image of a free-standing composite material in a tape-based holder (top). The scale bar is 2 cm. A representative top–down SEM image demonstrates that the overlaid copper coating is fractured and consists of multiple abutting domains (inset). The scale bar is 20 μm

We began our experiments by fabricating the desired thermoregulatory composite material according to the scheme in Fig. 2a. Here, we prepared samples with areas of >~160 cm^2^ by using common laboratory techniques and methods that would ultimately allow for larger-area manufacturing on an industrial scale. In brief, we electron-beam evaporated an infrared-reflecting, nanostructured copper film onto a support substrate via a two-step oblique angle deposition process (Fig. 2a, left)^33^, obtaining arrayed tilted nanoscale columns that emerged from an underlying continuous metal (copper) coating, as confirmed by top–down scanning electron microscopy (SEM) (Fig. 2b and Supplementary Fig. 1). Next, we spincast an infrared-transparent styrene–ethylene–butylene–styrene (SEBS) copolymer^34,35^ directly onto the nanostructured metal layer (Fig. 2a, middle), stably anchoring the copper columns within this elastomer, as confirmed by cross-sectional SEM (Fig. 2c and Supplementary Fig. 1). Subsequently, we heat-treated and delaminated the resulting composite from the support substrate to obtain a free-standing material (Fig. 2a, right), which featured a fractured, multi-domain copper coating on one side, as confirmed by top–down SEM (Fig. 2d). Overall, the described robust, high-yield procedure furnished a uniform and relatively large-area composite analogous to the one envisioned in Fig. 1e, f.

### Infrared and mechanical characterization

**Fig. 3:**
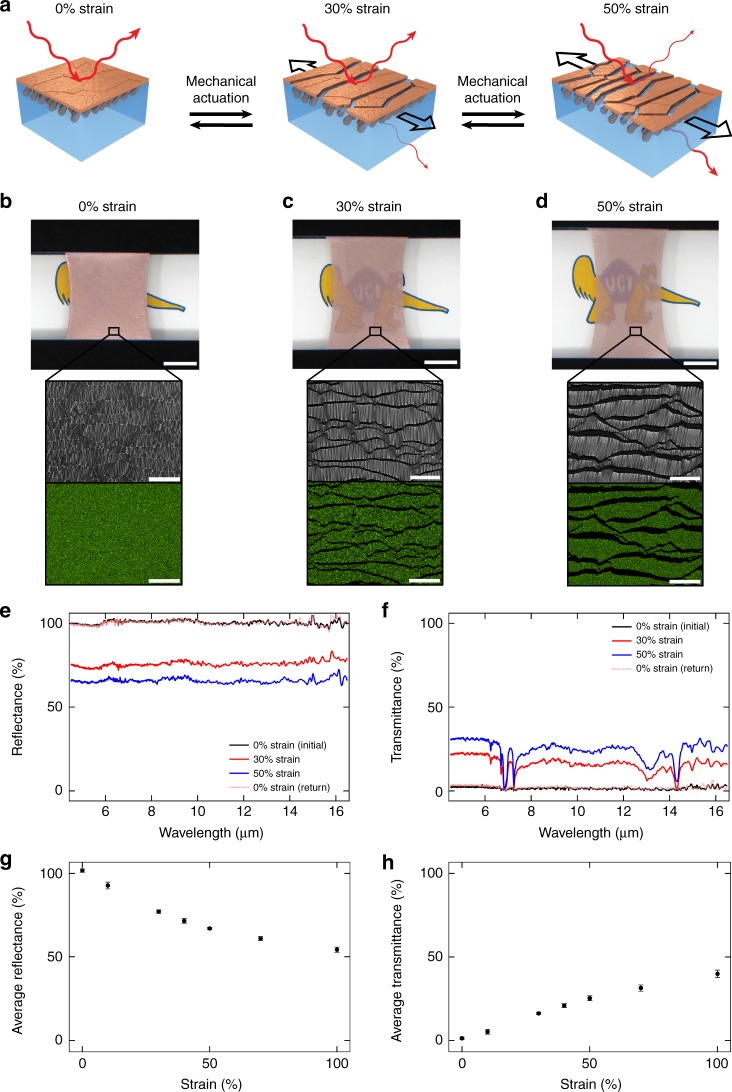
Mechanical actuation of changes in surface microstructure and infrared properties for the composite material. **a** Schematic of the mechanical actuation of the composite with strains of 0% (left), 30% (middle), and 50% (right). The surface microstructure and the reflection and transmission of infrared radiation change as a function of the applied strain. **b** Digital camera image of a composite under a strain of 0% above an anteater cartoon (top). The scale bar is 1 cm. A top–down scanning electron microscopy (SEM) image and copper elemental map, where the metal is colored green, for the surface of a representative composite material under a strain of 0% (inset). The scale bars are 100 μm. **c** Digital camera image of a composite under a strain of 30% above an anteater cartoon (top). The scale bar is 1 cm. A top–down SEM image and copper elemental map, where the metal is colored green, for the surface of a representative composite material under a strain of 30% (inset). The scale bars are 100 μm. **d** Digital camera image of a composite under a strain of 50% above an anteater cartoon (top). The scale bar is 1 cm. A top–down SEM image and copper elemental map, where the metal is colored green, for the surface of a representative composite material under a strain of 50% (inset). The scale bars are 100 μm. **e** The total infrared reflectance spectra for a representative composite material under strains of 0% (black trace), 30% (red trace), and 50% (blue trace). The reflectance observed at 0% strain is recovered even after successive actuation with higher strains (pink dotted trace). **f** The total infrared transmittance spectra for a representative composite material under strains of 0% (black trace), 30% (red trace), and 50% (blue trace). The transmittance observed at 0% strain is recovered even after successive actuation with higher strains (pink dotted trace). **g** Plot of the decrease in the average total reflectance for representative composites as a function of the applied strain. All error bars represent the standard deviation. **h** Plot of the increase in the average total transmittance for representative composites as a function of the applied strain. All error bars represent the standard deviation

We initially characterized our composites’ mechanical properties via tensile testing, while also evaluating their surface microstructure at different applied strains. For our composites, the engineering stress vs. engineering strain curves revealed that they behaved like soft and stretchable elastomers, with an average elastic modulus of ~2 MPa and an elongation at break of ~700% (Supplementary Fig. 2). In their relaxed state, the composites were visibly opaque and highly reflective, as indicated by digital camera imaging, and their surfaces consisted of a dense arrangement of abutting, irregularly shaped metal domains, which completely covered the underlying polymer matrix, as confirmed by SEM and energy dispersive spectroscopy (EDS) (Fig. 3a, left and Fig. 3b). However, under a strain of 30%, the composites became partially transparent and less reflective, as indicated by digital camera imaging, and their surfaces consisted of a sparser arrangement of metal domains that were proximal to but not directly contacting one another, revealing some of the underlying strained polymer matrix, as confirmed by SEM and EDS (Fig. 3a, middle and Fig. 3c). Under a larger strain of 50%, the composites further increased their visual transparency and appeared even less reflective, as indicated by digital camera imaging, and their surfaces consisted of an arrangement of metal domains that were spread farther apart from each other, uncovering even more of the underlying strained polymer matrix, as confirmed by SEM and EDS (Fig. 3a, right and Fig. 3d). Importantly, the composites could be loaded/unloaded in <1 s, featured fully reversible changes in both their visible appearance and surface microstructure, and readily withstood repeated mechanical cycling. Interestingly, the composites demonstrated properties similar to those reported for various squid skin components, including elastic moduli (~0.5–3 MPa for the chromatophore-containing dermal layer)^25^, response times (<1 s for chromatophore organs)^26^, and capacities for extreme elongation (>14-fold diameter expansion for chromatophore pigment cells)^27^. Therefore, the composites exhibit several advantageous characteristics that are desired for thermal switching applications, including a rapid response time, a soft and stretchable form factor, and a straightforward actuation mechanism (Supplementary Table 2).

We next showed that the composites’ infrared reflectance and transmittance could be dynamically modulated on demand by an applied mechanical stimulus (a uniaxial strain). In its relaxed state, a representative composite featured a high average total reflectance of ~100% (Fig. 3e) and a low average total transmittance of ~1%, as well as no apparent signals associated with specific functional groups (Fig. 3f), due to complete coverage of the infrared-transparent polymer matrix by the infrared-reflecting copper coating (Fig. 3b). However, under a strain of 30%, the representative composite featured a reduced average total reflectance of ~77% (Fig. 3e) and an increased average total transmittance of ~16%, as well as signals corresponding to the chemical functionalities of SEBS (e.g., –C=C–, –CH_2_–, and =C–H) (Fig. 3f), confirming that the underlying polymer matrix was partially uncovered (Fig. 3c)^36,37^. Under an increased strain of 50%, the representative composite featured an even smaller average total reflectance of ~67% (Fig. 3e) and an even larger average total transmittance of ~25%, as well as increased intensities for the SEBS-associated spectroscopic signals (Fig. 3f), due to additional exposure of the underlying polymer matrix (Fig. 3d). In general, the composites’ reflectance and transmittance both demonstrated a non-linear dependence on the strain, with the reflectance decreasing by ~25 ± 1%, ~35 ± 1%, and ~47 ± 2% at strains of 30%, 50%, and 100%, respectively (Fig. 3g), and the transmittance increasing by ~15 ± 1%, ~24 ± 2%, and ~39 ± 3% at strains of 30%, 50%, and 100%, respectively (Fig. 3h) (all relative to the initial unstretched state). Notably, the composites displayed fully reversible changes in their infrared reflectance and transmittance (Fig. 3e, f) and also exhibited excellent stability, with no degradation in their functionality after >~10^3^ actuation cycles (Supplementary Fig. 3). Moreover, based on their transmittance at strains of 0% and 100%, our mechanically actuated materials possess maximum transmittance on/off ratios of >~25, which exceed the best values reported for radiative thermal switches (Supplementary Table 2). Thus, the composites showcase several additional characteristics desired for thermal switching applications, including reversibility, stability, and high on/off ratios (Supplementary Table 2).

### Thermal modeling and evaluation

**Fig. 4:**
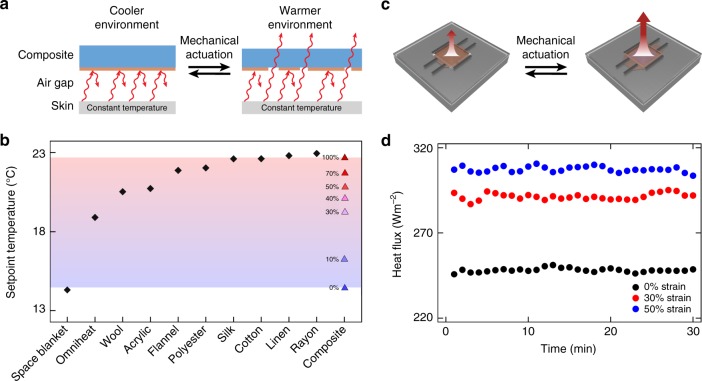
Mechanical control over the setpoint temperature and thermoregulatory properties for the composite material. **a** Schematic of the heat flux from human skin, through the composite material, and to a variable-temperature environment without (left) and with (right) mechanical actuation. The heat flux from the skin to the surroundings increases in order to maintain the skin temperature at a constant value upon going from a cooler (left) to a warmer (right) environment. **b** Plot of the environmental setpoint temperatures at which an individual would remain comfortable, i.e., maintain a constant skin temperature and unchanged outgoing heat flux, while wearing either various common types of cloth (black diamonds) or the composite material at different applied strains (blue and red triangles). The calculated setpoint temperature associated with the composite can be dynamically adjusted via mechanical actuation, and the composites’ accessible setpoint temperature range is indicated by the area shaded in red and blue. **c** Schematic of the heat flux from a sweating-guarded hot plate, through a composite, and to a controlled environment without (left) and with (right) mechanical actuation. **d** Plot of the steady-state heat flux from the hot plate as a function of time for a representative composite material under strains of 0% (black dots), 30% (red dots), and 50% (blue dots)

Having confirmed our composites’ tunable infrared properties, we evaluated their promise for adaptive thermal management within the context of a wearable configuration. To this end, we leveraged our spectroscopic measurements and computationally modeled heat transfer between human skin, the composite material, and the surrounding environment in various states of actuation (for comparison, we performed analogous experiments and calculations for several common types of cloth) (Fig. 4a, Supplementary Note 1, Supplementary Fig. 4, and Supplementary Table 3)^38,39^. We also determined the environmental setpoint temperatures, i.e., ones at which the body’s skin temperature and outgoing heat flux remain constant, that would ensure individuals maintained their thermal comfort while wearing the unstrained and strained composites (or types of cloth). In their relaxed state, the composites featured a setpoint temperature of ~14.5 °C, which was similar to the value of ~14.3 °C found for the space blanket (Fig. 4b). However, under a moderate strain of 30%, the composites featured a setpoint temperature of ~19.2 °C, which was similar to the value of ~18.9 °C found for a Columbia Omniheat fleece lining (Fig. 4b). In addition, under an increased strain of 50%, the composites featured a setpoint temperature of ~20.9 °C, which was similar to the value of ~20.5 °C found for wool (Fig. 4b). Moreover, under an even greater strain of 100%, the composites featured a setpoint temperature of ~22.7 °C, which was similar to the value of ~22.8 °C found for cotton (Fig. 4b). Taken together, our infrared spectroscopy experiments and subsequent calculations confirmed that the composites could be rapidly actuated with strain to maintain a wearer’s thermal comfort across an environmental setpoint temperature window of ~8.2 °C, which is among the largest dynamic ranges reported for any comparable passive material (Fig. 4b). In essence, the composites possess unprecedented switching capabilities, wherein their infrared reflectance and transmittance can be modulated in real time to emulate various types of cloth (note that common fabrics would typically exhibit small and/or irreversible changes in their infrared properties upon the application of strain). Assuming broad implementation in advanced garments that are widely deployed and adopted, our composites’ significant dynamic temperature setpoint window of >8 °C could enable an estimated reduction in building energy consumption of >30%^9,40,41^.

We next proceeded to validate our composites’ ability to adaptively manage heat transfer from a surface that emulated the thermal behavior of human skin. To this end, we mounted the composites in a custom-designed holder that allowed for the application of strain on a sweating guarded hot plate and then tested their thermoregulatory functionality under controlled conditions without and with mechanical actuation (Fig. 4c and Supplementary Fig. 5). In its relaxed state, a representative composite maintained the steady state heat flux from the hot plate at an average value of ~248 W m^−2^ (Fig. 4d). Under a moderate strain of 30%, the same composite maintained the steady state heat flux from the hot plate at a higher average value of ~291 W m^−2^ (Fig. 4d). Under a further increased strain of 50%, the composite maintained the steady state heat flux from the hot plate at an even higher average value of ~307 W m^−2^ (Fig. 4d). In general, the heat flux could be stably, reliably, and repeatedly modulated by average values of ~36 ± 8 and ~51 ± 8 W m^−2^ upon actuation with applied strains of 30% and 50%, respectively (with a maximum change of ~59 W m^−2^). Excitingly, based on these measurements, the composites can manage up to one quarter of the metabolic heat generation from the human body, which is expected to be ~70 W m^−2^ for a sedentary individual (and potentially much higher depending on the type of activity)^38,42^. In addition, the composites need an estimated input of only ~3 W m^−2^ for one mechanical switching event (at moderate strain) and do not continuously consume power, making their energy requirements substantially lower than all known active thermal management platforms (Supplementary Note 2 and Supplementary Table 1). Consequently, the composites demonstrate the key advantages of both passive and active systems by exhibiting excellent energy efficiency while simultaneously allowing for real-time user control.

### Human subject testing

**Fig. 5:**
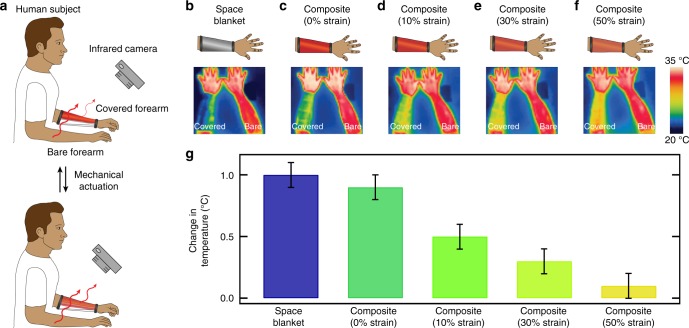
Adaptive localized regulation of body temperature with a mechanically actuated composite-based sleeve. **a** Schematic of the infrared camera-based visualization of the outgoing heat flux and local temperature for a human subject’s sleeve-covered forearm before mechanical actuation (top) and for the same human subject’s sleeve-covered forearm after mechanical actuation (bottom). The measurements are also performed for the wearer’s bare forearm during the experiment. **b** Schematic of a forearm covered with a space blanket-based sleeve (top). Infrared camera image of a forearm covered with a space blanket-based sleeve and a forearm that is bare (bottom). **c** Schematic of a forearm covered with a composite-based sleeve under a strain of 0% (top). Infrared camera image of a forearm covered with a composite-based sleeve under a strain of 0% and a forearm that is bare (bottom). **d** Schematic of a forearm covered with a composite-based sleeve under a strain of 10% (top). Infrared camera image of a forearm covered with a composite-based sleeve under a strain of 10% and a forearm that is bare (bottom). **e** Schematic of a forearm covered with a composite-based sleeve under a strain of 30% (top). Infrared camera image of a forearm covered with a composite-based sleeve under a strain of 30% and a forearm that is bare (bottom). **f** Schematic of a forearm covered with a composite-based sleeve under a strain of 50% (top). Infrared camera image of a forearm covered with a composite-based sleeve under a strain of 50% and a forearm that is bare (bottom). **g** Plot of the increase in temperature for a forearm covered with a space blanket-based sleeve and a forearm covered with a composite-based sleeve under strains of 0% 10%, 30%, and 50%, all relative to the increase in temperature measured simultaneously for a bare forearm. All error bars represent the standard deviation

Last, we assessed the composites’ ability to function as the dynamic switchable components in wearable systems and, more specifically, to locally manage the temperature of the human body. For this purpose, we fabricated custom-designed composite-based sleeves, which allowed for mechanical actuation of the joined composites via a straightforward fastener assembly (for comparative purposes, we prepared analogous space blanket-based sleeves) (Fig. 5a and Supplementary Fig. 6). Subsequently, we outfitted a human subject with these sleeves and then recorded the outgoing heat flux and apparent change in temperature for both the sleeve-covered and bare forearms of the wearer, while actuating the composites with different strains (note that the forearms served as internal standards that facilitate comparisons across all measurements) (Fig. 5b–f and Supplementary Fig. 7). In initial benchmark experiments, a static space blanket-based sleeve trapped (primarily reflected back) the heat emitted by the covered forearm (Fig. 5b) and thus raised its temperature by ~1.0 ± 0.1 °C more than the bare forearm of the same person (Fig. 5g and Supplementary Fig. 7). Similarly, without applied strain, the composite-based sleeve trapped the heat emitted by the covered forearm (Fig. 5c) and raised its temperature by ~0.9 ± 0.1 °C more than the same person’s bare forearm (Fig. 5g and Supplementary Fig. 7), much like the space blanket. However, for an applied strain of 30%, the composite-based sleeve trapped only some of the heat emitted by the covered forearm (Fig. 5e) and raised its temperature by only ~0.3 ± 0.1 °C more than the same person’s bare forearm (Fig. 5g and Supplementary Fig. 7), which constituted a >2-fold reduction in the temperature change measured for the space blanket. Furthermore, for an applied strain of 50%, the composite-based sleeve trapped substantially less of the heat emitted from the covered forearm (Fig. 5f) and raised its temperature by only ~0.1 ± 0.1 °C more than the same person’s bare forearm (Fig. 5g and Supplementary Fig. 7), which represented a ~10-fold reduction in the temperature change measured for the space blanket. Overall, the measurements demonstrated that the composite-based sleeves could be adjusted to manage the heat flux from the wearer to the surrounding environment in real time (Fig. 5b–f and Supplementary Movie 1) and to modulate the wearer’s local changes in body temperature by nearly an order of magnitude with excellent control (Fig. 5g). Importantly, the maximum measured temperature changes were ~2–5-fold greater than the reported temperature perception thresholds for a variety of human subjects, allowing users of the composite-based sleeves to readily perceive the thermal effect of the different actuation states^43^. In principle, the performance of the sleeves and comfort of the wearer could be improved further through the use of composites from comparable infrared-transparent polymer matrices with enhanced breathabilities. Overall, these exciting preliminary findings lay the groundwork for the development of sophisticated composite material-based garments, which can be site-specifically actuated via more advanced strategies and thus allow for regulation of the local thermal environment across a wearer’s entire body.

## Discussion

In summary, we have developed an artificial thermoregulatory platform that leverages the static infrared-reflecting design of the space blanket and draws inspiration from the dynamic color-changing ability of squid skin, and our findings hold significance for several reasons. First, our composites function via a unique but generalizable mechanism that relies on reversible, mechanically actuated changes in surface microstructure and, consequently, can be further optimized through the use of alternative infrared-reflecting metals/oxides, e.g., titanium dioxide, or other elastic infrared-transparent polymers, e.g., polyethylene derivatives. Second, these stimuli-responsive materials alter their reflectance and transmittance within the thermal infrared region of the electromagnetic spectrum and thus can adjust their thermoregulatory properties to resemble those of other common wearable materials, such as the space blanket, an Omniheat fleece lining, wool, and cotton. Third, our composites essentially behave like radiative thermal switches and possess a highly desirable combination of metrics, including ease of manufacturing over large areas, a straightforward actuation method, reversibility and tunability without hysteresis, stability to repeated cycling, a soft and stretchable form factor, a rapid response time, a high on/off ratio, and a low operating temperature (Supplementary Table 2). Fourth, our materials can manage the trapping/release of a significant portion of the thermal flux expected from a resting individual, while requiring only a fraction of this energy for actuation, and thereby serve as an energy-efficient conceptual and technological bridge between passive and active approaches to thermal management. Fifth, our composites possess one of the largest reported dynamic environmental setpoint temperature ranges and can precisely regulate local body temperature changes for wearers in real time, indicating that they may be appropriate for integration into more advanced personal thermal management systems that are electrically or electromechanically actuated. Last, our materials are manufactured from low-cost commercial building blocks via scalable and facile processes, portending favorably for their use in a great variety of applications, ranging from traditional ones, such as clinical warming devices and shipping containers, to emerging ones, such as conformable electronic skin and untethered soft robots. Overall, our bioinspired platform features unprecedented capabilities and figures of merit, which dramatically advance the state of the art in multiple areas, and as such, it holds the potential to shift modern energy consumption paradigms upon widespread implementation.

## Methods

### Fabrication of the composite materials

The composite materials were produced according to standard microfabrication protocols. First, to prepare the nanostructured layer, a ~20-nm planar copper (Kurt J. Lesker) film and a ~90-nm array of tilted columns were sequentially electron beam evaporated onto a 6-inch diameter silicon wafer (University Wafer) by using an Angstrom Engineering EvoVac system. Next, to embed the nanostructured layer within an infrared-transparent elastomer, a ~30-μm-thick film from a commercially available SEBS block copolymer (G1645 SEBS, Kraton Polymers LLC) was spincast directly onto the nanostructure-modified substrate. Last, the composite was heat-treated on a hot plate at 60 °C for 10 min and then delaminated from the substrate by means of a Mylar frame and pressure-sensitive tape. The resulting materials obtained via this procedure were used for physical, mechanical, optical, and infrared characterization experiments as needed.

### SEM of the composite materials

The morphology of the composite materials was characterized via SEM by using a Magellan 400 XHR SEM (FEI). For imaging of the nanostructured layer, the pristine samples were not modified. For cross-sectional imaging of the interior of the composite materials, a portion of the elastomer was removed with a Hitachi IM4000 Plus Ion Milling System. For imaging of the copper domains at various strains, the samples were subjected to the appropriate strain, fixed with an epoxy resin (Ted Pella), and sputter coated with an ~2-nm film of platinum/palladium on a Leica ACE200 Vacuum Coater. Such characterization ensured quality control and standardization during the fabrication and testing steps.

### Elemental mapping of the composite materials

The chemical composition of the materials’ surfaces was characterized via EDS by using a Magellan 400 XHR SEM outfitted with an Oxford Instruments X-Max Silicon Drift Detector. For these measurements, the samples were again subjected to various strains, fixed with an epoxy resin, and sputter coated with an ~2-nm film of platinum/palladium. The resulting copper elemental maps were analyzed by using the ImageJ (NIH) software package and were consistent with expectations from the SEM experiments. Such characterization further confirmed the chemical identity of the copper domains covering the surface of the elastomer.

### Infrared spectroscopy of the composite materials and cloths

The infrared properties of the composite materials and various types of cloth were characterized according to established protocols^44^ via infrared spectroscopy by using a Perkin Elmer Fourier transform infrared (FTIR) Spectrometer outfitted with a Pike Technologies Mid-infrared Integrating Sphere. The measurements furnished the total (sum of diffuse and specular) reflectance and transmittance and were referenced to a Pike Technologies Diffuse Gold Standard, unless otherwise specified. During the experiments, all samples were mounted on home-built size-adjustable stages, which allowed for mechanical actuation, i.e., the application of different uniaxial strains, as necessary. The samples tested included rayon, cotton, linen, silk, polyester, cotton flannel, acrylic, wool, the Columbia Omniheat fleece, the space blanket, and the composite material, and the tested areas were large enough to completely cover the ~2 cm diameter instrument port before and after mechanical actuation. The total transmittance spectra were collected at a normal illumination angle, and the total reflectance spectra were collected at an illumination angle of 12°. The collected spectra were analyzed with the Spectrum (PerkinElmer) and Igor Pro (Wavemetrics) software packages, and all average values were calculated over the wavelength range of 4.5–16.5 μm. The experiments were performed for at least ten different samples, with similar results obtained in each instance.

### Tensile testing of the composite materials

The tensile properties of the composite materials were characterized according to standard protocols^45^ by using an Instron 3365 Universal Testing System. During the experiments, samples with a 3-inch length, 0.5-inch width, and 30 μm thickness were mounted on the grips of the instrument at an initial separation of 1 inch. The measurements furnished the load vs. the elongation, enabling calculation of the engineering stress as a function of the engineering strain. The engineering stress (*σ*) was calculated according to the equation:

1 $$\sigma = F/A$$

where *F* is the applied load and *A* is the area of the sample. The engineering strain (*ε*) was calculated according to the equation:

2 $$\varepsilon = {\Delta }L/L$$

where Δ*L* is the change in length of the sample and *L* is the initial length of the sample. The Young’s modulus (*E*) was calculated from the linear region of the engineering stress vs. the engineering strain (at low strain) by using the following equation:

3 $$E = \sigma /\varepsilon$$

where *E* is the slope of the elastic (linear) region. The experiments were performed for at least ten different samples, with similar results obtained in each instance.

### Stability testing of the composite materials

The mechanical stability of the composite materials was characterized by using a MARK-10 ESM303 Tension/Compression Test Stand, in conjunction with a Perkin Elmer FTIR Spectrometer outfitted with a Pike Technologies Mid-Infrared Integrating Sphere. First, the samples’ total infrared reflectance and transmittance were measured at uniaxial strains of 0%, 30%, and 50%, as described above. Next, the samples were cycled 1000 times between applied strains of 0% and 50%. In turn, the samples’ total infrared reflectance and transmittance were again measured at uniaxial strains of 0%, 30%, and 50%, as described above. The experiments were performed for at least four different samples, with similar results obtained in each instance.

### Thermal evaluation of the composite materials

The thermal properties of the composite materials were characterized according to standard protocols^46^ by using a Thermetrics SGHP-8.2 Sweating Guarded Hot Plate located within a temperature- and humidity-controlled chamber (Supplementary Fig. 5). During the experiments, all samples were mounted on custom-designed holders, which allowed for them to be maintained under different applied uniaxial strains on the hot plate. The measurements furnished the thermal flux from the hot plate necessary to maintain its temperature at a constant value, as well as the thermal resistance of the sample being tested. For the measurements, the hot plate was covered with skin tape (Trimaco) and kept at a human skin-like temperature of 35 °C; the surrounding chamber was maintained at an environmental temperature of 19.5 °C and a relative humidity of 50%; and the sample was maintained under a laminar air flow of 1 m s^−1^. The average values of the thermal flux were calculated from multiple independent measurements performed over a period of 30 min for both strained and unstrained samples. The collected data were analyzed with the Igor Pro (Wavemetrics) software package. The experiments were performed for at least three different samples, with similar results obtained in each instance.

### Fabrication of the wearable sleeves

The integrated sleeves, which consisted of a composite-based adaptive component and a fabric-based actuation component, were designed and manufactured via protocols developed in-house (Supplementary Fig. 6). To prepare the composite-based adaptive component, four circular samples of the composites with an ~6-inch diameter were first segmented into rectangular pieces with an ~5.5-inch length and an ~2.5-inch width. The four rectangular pieces were then joined together with adhesive tape to form a larger section with an ~10-inch length and an ~5.5-inch width. The larger section was in turn modified with hook fasteners (from a commercial hook-and-loop fastener set) along its periphery, in order to enable integration with the fabric-based component and allow for the application of different strains. To prepare the fabric-based actuation component, a square sample of a stretch woven fabric was first cut into a sleeve-like pattern. The resulting fabric swatch was then modified with loop fasteners (from the same commercial hook-and-loop fastener set) at predetermined positions, which were selected to correspond to the application of strains of 0%, 10%, 30%, and 50% to the composite-based component. The fabric swatch was in turn modified with support bands, which encircled the forearm to provide structural support and simultaneously functioned as rail guides to prevent the composite-based component from directly contacting the skin. To complete the wearable adaptive thermal management sleeve, the fabric-based actuation component was mounted on the forearm of a human subject, and the composite-based adaptive component was then attached to its fabric-based counterpart via the complementary hook-and-loop fastener assembly. Here note that the space blanket-based sleeves were prepared by following nearly identical protocols to those described above. All variants of the sleeves were used for thermal management experiments with human subjects immediately after fabrication.

### Human subject testing of the wearable sleeves

The thermoregulatory properties of the composite-based sleeves were evaluated for human subjects by using a FLIR C2 Infrared Camera. First, the bare forearms of a human subject were allowed to equilibrate in a controlled environment, and both forearms were imaged to record their average initial temperature. Next, the composite-based sleeve was mounted on one of the forearms, with the adaptive composite-based component attached at a loop fastener position corresponding to the absence of strain, and both the bare and sleeve-covered forearms were allowed to equilibrate for a period of 10 min. In turn, the composite-based sleeve was removed from the previously covered forearm, and both forearms were imaged again to record their final average temperature. Last, the same sequence of measurements was repeated with the adaptive composite-based component attached at a loop fastener position corresponding to different applied strains of ~10%, ~30%, and ~50%. Note that the sleeve removal and measurement steps were performed sufficiently rapidly to minimize effects from cooling of the skin of the bare forearm, which cools slowly in air from ~33 °C to ~28 °C over a span of 120 min^47^. From these measurements, the relative change in temperature (Δ*T*) for the human subject’s sleeve-covered forearm with respect to his/her bare forearm, which served as a de facto internal standard, was calculated according to the equation:

4 $${\Delta }{T} = {\Delta }{T}_{{\mathrm{covered}}}-{\Delta }{T}_{{\mathrm{bare}}} = ( {T_{{\mathrm{covered,final}}}-T_{{\mathrm{covered,initial}}}} ) - ( {T_{{\mathrm{bare,final}}}-T_{{\mathrm{bare,initial}}}} )$$

where Δ*T*_covered_ is the average temperature change for the covered forearm, Δ*T*_bare_ is the average temperature change for the bare forearm, *T*_covered,final_ is the average final temperature for the covered forearm, *T*_covered,initial_ is the average initial temperature for the covered forearm, *T*_bare,final_ is the average final temperature for the bare forearm, and *T*_bare,initial_ is the average initial temperature for the bare forearm. For the presented measurements, the human subject was a sedentary 24-year-old male for whom overall perspiration remained relatively unchanged during the course of any specific experiment, and the surrounding room temperature was maintained at ~17.5–18.5 °C. The average forearm temperatures were calculated directly from the obtained videos and images by using the FLIR Tools+ (FLIR) and ImageJ (NIH) software packages. The calculation of the average temperature from the infrared camera images over the entire forearm mitigated the influence of unexpected temperature fluctuations and hot and cold spots on our analysis^48^, with complementary thermal couple measurements providing additional confirmation and validation for the observed trends. Here the thermoregulatory properties of the space blanket-based sleeves were evaluated by following nearly identical protocols to those described above. All variants of the experiments were performed for at least seven different sleeves, with similar results obtained in each instance.

### Human subjects' research

Informed consent was obtained from the human subjects who participated in the research. The human subject testing protocol was reviewed and approved by the UC Irvine Institutional Review Board (IRB).

## Supplementary information

Supplementary Information

Supplementary Movie

Description of Additional Supplementary Files

## Data Availability

All data needed to evaluate the conclusions in the paper are present in the paper and/or the supplementary materials.
