# Supplementary material for: A dynamic thermoregulatory material inspired by squid skin
Source: Nat Commun. 2019 Apr 29;10:1947. doi: 10.1038/s41467-019-09589-w (PMC6488639; doi:10.1038/s41467-019-09589-w)
Supplement: Supplementary file 1 — Supplementary Information [file 41467_2019_9589_MOESM1_ESM.pdf]

A Dynamic Thermoregulatory Material Inspired by Squid Skin

Leung et al.

Supplementary Information

Supplementary Note 1. Analysis of heat transfer through the composite materials or cloth.

The thermal properties of the composite materials were computationally evaluated according to procedures adapted from the literature[1,2]. Specifically, we calculated the environmental setpoint temperature (T_e), which is defined as the temperature of the surroundings at which a sedentary individual remains comfortable, i.e. maintains a constant skin temperature of 35 °C and keeps a steady state heat generation of 73 Wm^{-2} [1]. For this purpose, the steady state, one-dimensional heat transfer (via conduction, convection, and radiation) between a heat source that emulates human skin, the composite or cloth, and the surrounding environment was modeled for the general configuration shown in Supplementary Fig. 4a according to the equations listed below.

The absorptance (α) was calculated according to the equation:

$$\alpha = 1 - \tau - \rho \quad (1)$$

where τ is the transmittance and ρ is the reflectance.

The emittance (ϵ) was calculated using Kirchoff's Law of Thermal Radiation according to the equation:

$$\epsilon = \alpha = 1 - \tau - \rho \quad (2)$$

where α is the absorptance, τ is the transmittance, and ρ is the reflectance.

The heat transferred from the skin via radiation ($q_{\text{rad,s}}$) was calculated using the Stefan-Boltzmann Law according to the equation:

$$q_{\text{rad,s}} = \epsilon_{\text{s}} \sigma T_{\text{s}}^4 \quad (3)$$

where σ is the Stefan-Boltzmann constant, ϵ_{s} is the emittance of the skin, and T_{s} is the temperature of the skin.

The heat transferred from the environment via radiation ($q_{\text{rad,e}}$) was calculated using the Stefan-Boltzmann Law according to the equation:

$$q_{\text{rad,e}} = \epsilon_{\text{e}} \sigma T_{\text{e}}^4 \quad (4)$$

where σ is the Stefan-Boltzmann constant, ϵ_{e} is the emittance of the environment, and T_{e} is the temperature of the environment.

The heat transferred from the inner side of the composite/cloth via radiation ($q_{\text{rad,i}}$) was calculated using the Stefan-Boltzmann Law according to the equation:

$$q_{\text{rad,i}} = \epsilon_{\text{i}} \sigma T_{\text{i}}^4 \quad (5)$$

where σ is the Stefan-Boltzmann constant, ϵ_{i} is the emittance of the inner side of the composite/cloth, and T_{i} is the temperature of the inner side of the composite/cloth.

The heat transferred from the outer side of the composite/cloth via radiation ($q_{\mathrm{rad,o}}$) was calculated using the Stefan-Boltzmann Law according to the equation:

$$q_{\mathrm{rad,o}} = \epsilon_{\mathrm{o}} \sigma T_{\mathrm{o}}^4 \quad (6)$$

where σ is the Stefan-Boltzmann constant, ϵ_{o} is the emittance of the outer side of the composite/cloth, and T_{o} is the temperature of the outer side of the composite/cloth.

The heat transferred through the air gap via conduction ($q_{\mathrm{cond,a}}$) was calculated using Fourier's Law according to the equation:

$$q_{\mathrm{cond,a}} = \frac{k_{\mathrm{a}}}{t_{\mathrm{a}}}(T_{\mathrm{s}} - T_{\mathrm{i}}) \quad (7)$$

where k_{a} is the thermal conductivity of air, t_{a} is the thickness of the air gap, T_{s} is the temperature of the skin, and T_{i} is the temperature of the inner side of the composite/cloth.

The heat transferred to the environment via convection (q_{conv}) was calculated using Newton's Law of Cooling according to the equation:

$$q_{\mathrm{conv}} = h(T_{\mathrm{o}} - T_{\mathrm{e}}) \quad (8)$$

where h is the convective heat transfer coefficient, T_{o} is the temperature of the outer side of the composite/cloth, and T_{e} is the temperature of the environment.

The total heat transferred from the skin ($q_{\mathrm{out,s}}$), including contributions from incident heat not absorbed by the skin, was calculated according to the equation:

$$q_{\mathrm{out,s}} = q_{\mathrm{rad,s}} + (1 - \epsilon_{\mathrm{s}}) \left(\frac{q_{\mathrm{rad,i}} + \rho_{\mathrm{i}} q_{\mathrm{rad,s}} + \tau_{\mathrm{c}} q_{\mathrm{rad,e}}}{1 - \rho_{\mathrm{i}}(1 - \epsilon_{\mathrm{s}})} \right) \quad (9)$$

where $q_{\mathrm{rad,s}}$ is the heat transferred from the skin via radiation, ϵ_{s} is the emittance of the skin, $q_{\mathrm{rad,i}}$ is the heat transferred from the inner side of the composite/cloth via radiation, ρ_{i} is the reflectance from the inner side of the composite/cloth, τ_{c} is the transmittance of the composite/cloth, and $q_{\mathrm{rad,e}}$ is the heat transferred from the environment via radiation.

The energy balance for the skin was calculated according to the equation:

$$q_{\mathrm{gen}} - (1 - \rho_{\mathrm{i}}) q_{\mathrm{out,s}} + q_{\mathrm{rad,i}} + \tau_{\mathrm{c}} q_{\mathrm{rad,e}} - q_{\mathrm{cond,a}} = 0 \quad (10)$$

where q_{gen} is the heat flux generated from the human body, ρ_{i} is the reflectance on the inner side of the composite/cloth, $q_{\mathrm{out,s}}$ is the total heat transferred from the skin, $q_{\mathrm{rad,i}}$ is the heat transferred from the inner side of the composite/cloth via radiation, τ_{c} is the transmittance of the composite/cloth, $q_{\mathrm{rad,e}}$ is the heat transferred from the environment via radiation, and $q_{\mathrm{cond,a}}$ is the heat transferred across the air gap via conduction.

The energy balance for the composite/cloth was calculated according to the equation:

$$(1-\rho_{\mathrm{i}}-\tau_{\mathrm{c}})q_{\mathrm{rad,s}}+(1-\rho_{\mathrm{o}}-\tau_{\mathrm{c}})q_{\mathrm{rad,e}}-q_{\mathrm{rad,o}}-q_{\mathrm{rad,i}}+q_{\mathrm{cond,a}}-q_{\mathrm{conv}}=0 \quad (11)$$

where ρ_{i} is the reflectance on the inner side of the composite/cloth, τ_{c} is the transmittance of the composite/cloth, $q_{\mathrm{rad,s}}$ is the heat transferred from the skin via radiation, ρ_{o} is the reflectance on the outer side of the composite/cloth, $q_{\mathrm{rad,e}}$ is the heat transferred from the environment via radiation, $q_{\mathrm{rad,o}}$ is the heat transferred from the outer side of the composite/cloth via radiation, $q_{\mathrm{rad,i}}$ is the heat transferred from the inner side of the composite/cloth via radiation, $q_{\mathrm{cond,a}}$ is the heat transferred across the air gap via conduction, and q_{conv} is the heat transferred to the environment via convection.

The temperature profile across the composite/cloth ($T(x)$) was calculated according to the equation:

$$T(x)=\frac{1}{2k_{\mathrm{c}}t_{\mathrm{c}}}\left(q_{\mathrm{rad,i}}+q_{\mathrm{rad,o}}-\epsilon_{\mathrm{i}}q_{\mathrm{out,s}}-\epsilon_{\mathrm{o}}q_{\mathrm{rad,e}}\right)x^2-\frac{k_{\mathrm{a}}}{k_{\mathrm{c}}}\left(\frac{T_{\mathrm{s}}-T_{\mathrm{i}}}{t_{\mathrm{a}}}\right)x+T_{\mathrm{i}} \quad (12)$$

where k_{c} is the thermal conductivity of the composite/cloth, t_{c} is the thickness of the composite/cloth, $q_{\mathrm{rad,i}}$ is the heat transferred from the inner side of the composite/cloth via radiation, $q_{\mathrm{rad,o}}$ is the heat transferred from the outer side of the composite/cloth via radiation, ϵ_{i} is the emittance from the inner side of the composite/cloth, $q_{\mathrm{out,s}}$ is the total heat transferred from the skin, ϵ_{o} is the emittance from the outer side of the composite/cloth, $q_{\mathrm{rad,e}}$ is the heat transferred from the environment via radiation, k_{a} is the thermal conductivity of air, T_{s} is the temperature of the skin, T_{i} is the temperature of the inner side of the composite/cloth, and t_{a} is the thickness of the air gap.

The temperature of the outer side of the composite/cloth ($T_{\mathrm{o}}=T(t_{\mathrm{a}})$) was evaluated according to the equation:

$$T_{\mathrm{o}}=\frac{t_{\mathrm{c}}}{2k_{\mathrm{c}}}\left(q_{\mathrm{rad,i}}+q_{\mathrm{rad,o}}-\epsilon_{\mathrm{i}}q_{\mathrm{out,s}}-\epsilon_{\mathrm{o}}q_{\mathrm{rad,e}}\right)-\frac{k_{\mathrm{a}}t_{\mathrm{c}}}{k_{\mathrm{c}}t_{\mathrm{a}}}(T_{\mathrm{s}}-T_{\mathrm{i}})+T_{\mathrm{i}} \quad (13)$$

where t_{c} is the thickness of the composite/cloth, k_{c} is the thermal conductivity of the composite/cloth, $q_{\mathrm{rad,i}}$ is the heat transferred from the inner side of the composite/cloth via radiation, $q_{\mathrm{rad,o}}$ is the heat transferred from the outer side of the composite/cloth via radiation, ϵ_{i} is the emittance from the inner side of the composite/cloth, $q_{\mathrm{out,s}}$ is the total heat transferred from the skin, ϵ_{o} is the emittance from the outer side of the composite/cloth, $q_{\mathrm{rad,e}}$ is the heat transferred from the environment via radiation, k_{a} is the thermal conductivity of air, t_{a} is the thickness of the air gap, T_{s} is the temperature of the skin, and T_{i} is the temperature of the inner side of the composite/cloth.

From the above equations, the environmental setpoint temperature (T_{e}) was calculated for the composites under different applied strains and for various types of cloth. For the calculations, thermal comfort was defined as the conditions under which the total heat generation rate and total heat dissipation rate are equal. The environment was approximated as a black body with $\epsilon=1$,

and the skin was approximated as a gray body with $\epsilon = 0.98$. The average reflectance and transmittance values were calculated from experimental measurements, such as the ones shown in Supplementary Fig. 4b-d for the composite material. The average values for rayon, cotton, silk, linen, polyester, flannel, acrylic, wool, the Columbia Omniheat fleece, the space blanket, and the composite material at various strains are listed in Supplementary Table 3. For the purpose of the calculations, the average reflectance and average transmittance values for the composites and various types of cloth were normalized with respect to the highly-reflective space blanket as an internal standard, rather than to the Pike Technologies Diffuse Gold Standard. Such normalization was performed because the Diffuse Gold Standard, which is validated by NIST and widely accepted, has a lower total infrared reflectance than either a gold mirror or the mirror-like space blanket[3-5]. The thermal conductivities of the composites and various types of cloth were estimated from reported literature values, which are listed in Supplementary Table 3. The thicknesses of the composites under different applied strains were determined experimentally or calculated by using a Poisson's ratio of 0.5, and the thicknesses of the various types of cloth were experimentally measured with calipers. The general wear scenario was defined as a human subject covered with a typical garment, for which the air gap thickness was estimated as 5 mm and the convective heat transfer coefficient was estimated as ~ 7.3 Wm^{-2}. The application of strain primarily modulates the radiative component of the heat flux through the composite materials in this wear scenario. In the calculations for the composites, the space blanket, and the Columbia Omniheat fleece, the inner side facing toward the skin was the metal coating, and the outer side facing toward the external environment was the polymer/fabric. Based on these approximations, measurements, and definitions, the calculations yielded setpoint temperatures as shown in Supplementary Table 3.

Supplementary Note 2. Analysis of the power consumed during composite actuation.
The power consumption associated with actuation of the composite materials was estimated according to standard procedures. First, the energy density (U) for the composites was calculated from the stress versus strain curve according to the equation:

$$U = \int_{\varepsilon_{\mathrm{i}}}^{\varepsilon_{\mathrm{f}}} \sigma d\varepsilon$$

where σ is the stress, ε_{i} is the initial strain, and ε_{f} is the final strain.

Next, the power consumption (P) was calculated from the equation:

$$P = \frac{U}{t}$$

where t is the time required for actuation.

a b c

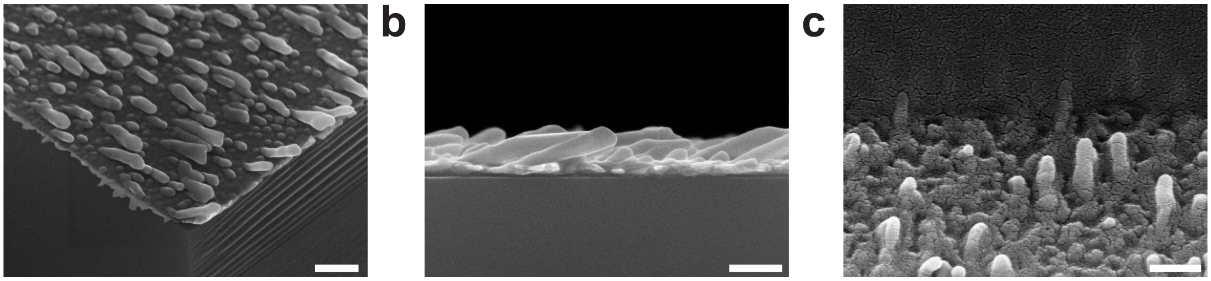

Supplementary Figure 1. Scanning electron microscopy images of copper nanostructures. a, A representative oblique-angle SEM image of a nanostructured copper film on a silicon dioxide/silicon substrate. The scale bar is 200 nm. **b**, A representative cross-sectional SEM image of a nanostructured copper film on a silicon dioxide/silicon substrate. The scale bar is 200 nm. **c**, A representative cross-sectional SEM image of copper nanostructures embedded within a polymer. The scale bar is 200 nm.

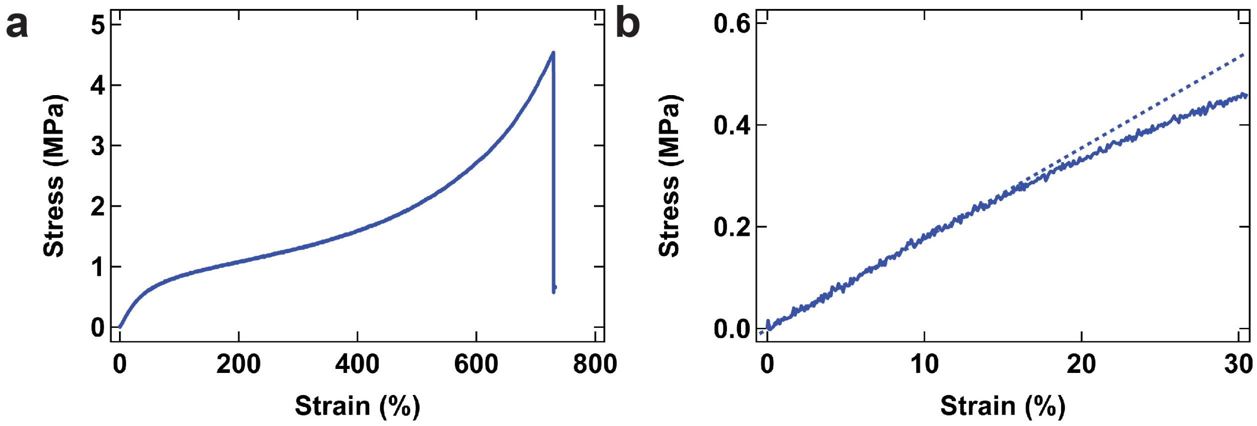

Supplementary Figure 2. Tensile testing of the composite material. a, A plot of the engineering stress versus the engineering strain for a representative composite material over the full tested strain range. **b**, A zoomed-in view of the low strain region of the plot in **a**. The dashed line represents a linear fit of the data, which was used to calculate the elastic modulus.

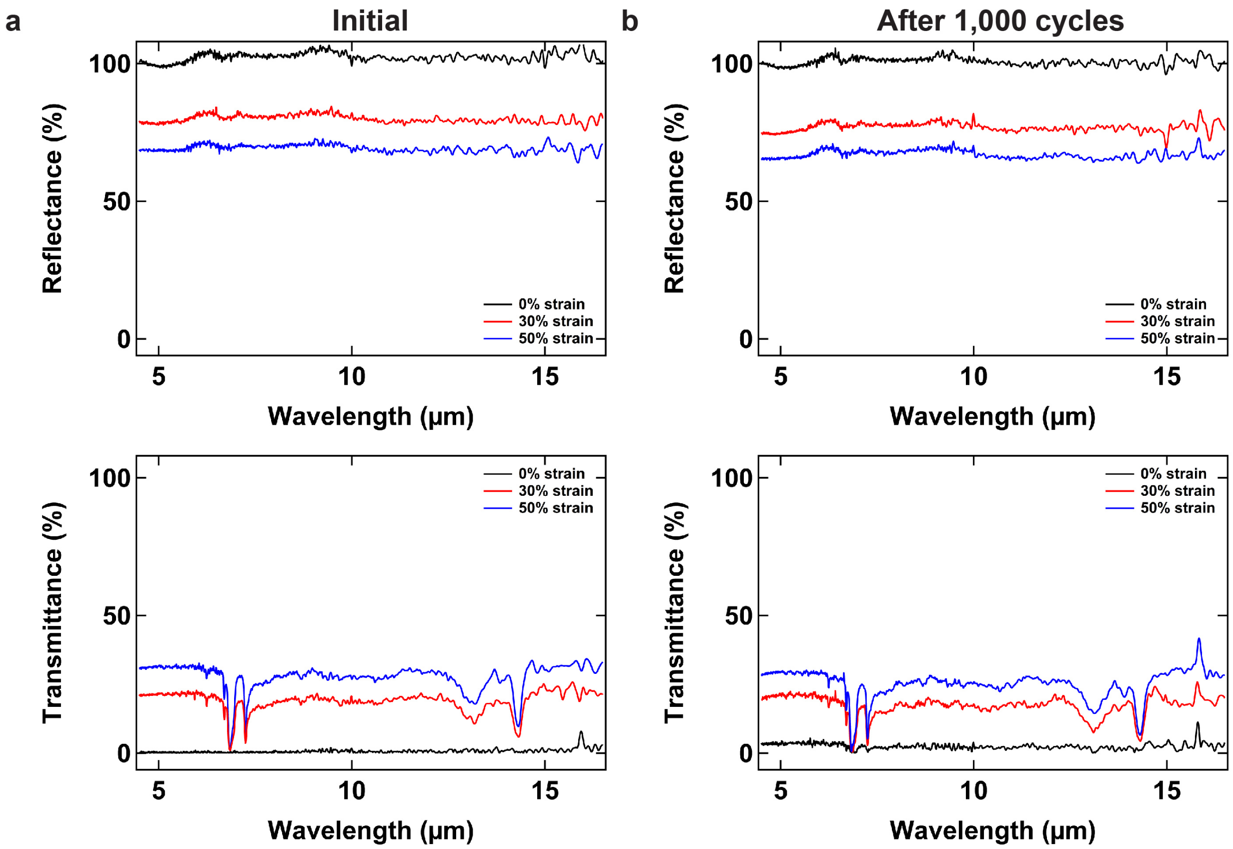

Supplementary Figure 3. Stability testing of the composite material. a, The infrared reflectance (top) and transmittance (bottom) spectra for a representative composite material under strains of 0 % (black trace), 30 % (red trace), and 50 % (blue trace). **b**, The infrared reflectance (top) and transmittance (bottom) spectra for the representative composite material from **a** under strains of 0 % (black trace), 30 % (red trace), and 50 % (blue trace) after 10^3 consecutive mechanical actuation cycles.

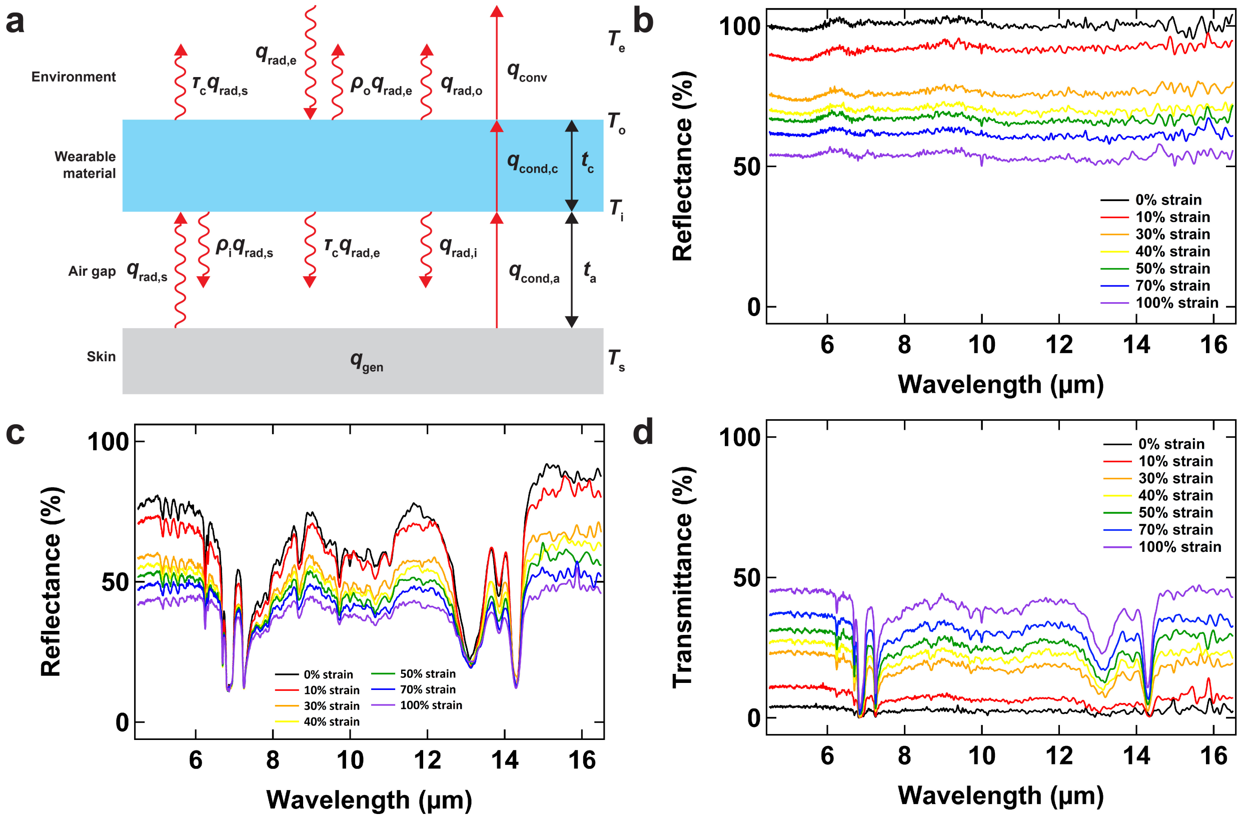

Supplementary Figure 4. Heat transfer through wearable materials and mechanical modulation of the infrared properties of the composite material. a, Schematic of the transfer of heat between human skin, a wearable material (such as the composite or various types of cloth), and the surrounding environment via radiation, convection, and conduction. **b**, The infrared reflectance spectra obtained for a representative composite material, with the infrared radiation incident on the copper-coated side (inner side), under strains of 0 % (black trace), 10 % (red trace), 30 % (orange trace), 40 % (yellow trace), 50 % (green trace), 70 % (blue trace), and 100 % (purple trace). **c**, The infrared reflectance spectra obtained for a representative composite material, with the infrared radiation incident on the polymer side (outer side), under strains of 0 % (black trace), 10 % (red trace), 30 % (orange trace), 40 % (yellow trace), 50 % (green trace), 70 % (blue trace), and 100 % (purple trace). **d**, The infrared transmittance spectra obtained for a representative composite material under strains of 0 % (black trace), 10 % (red trace), 30 % (orange trace), 40 % (yellow trace), 50 % (green trace), 70 % (blue trace), and 100 % (purple trace).

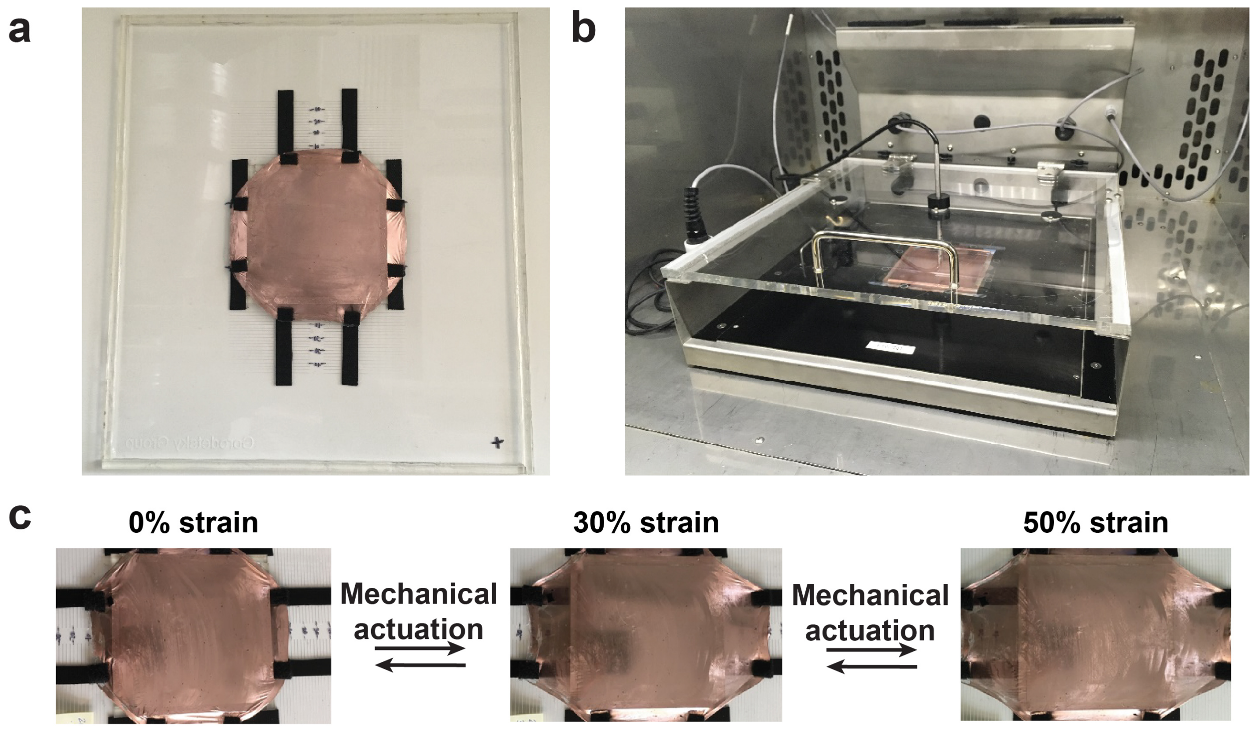

Supplementary Figure 5. Evaluation of the thermoregulatory properties of the mechanically-actuated composite material. a, Digital camera image of a representative composite mounted on a custom-designed holder that allows for the application of strain via hook-and-loop fasteners. **b**, Digital camera image of a representative holder-mounted composite on a sweating guarded hot plate within an environmentally-controlled chamber. **c**, Digital camera images of a representative holder-mounted composite under strains of 0 %, 30 %, and 50 %.

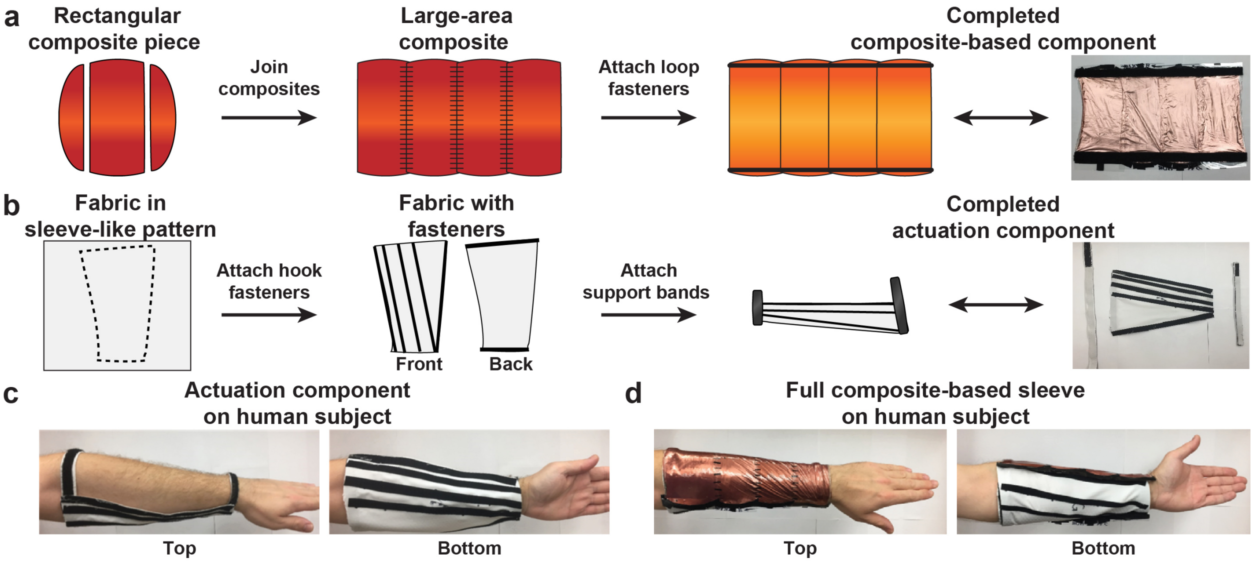

Supplementary Figure 6. Fabrication and donning of the composite-based sleeve. a, Schematic of the general fabrication procedure for the composite-based adaptive component. The steps consist of segmenting the circular composites into rectangular pieces, joining the pieces to form a larger composite section, and modifying the section with hook fasteners along its periphery. A digital camera image of the composite-based component is shown on the right. **b**, Schematic of the general fabrication procedure for the fabric-based actuation component. The steps consist of cutting a square stretch woven fabric sample into a sleeve-like pattern, modifying the resulting fabric swatch with loop fasteners at predetermined positions, and incorporating support bands. A digital camera image of the fabric-based component is shown on the right. **c**, Digital camera images of the top (left) and bottom (right) of the fabric-based actuation component mounted on the forearm of a human subject. **d**, Digital camera images of the top (left) and bottom (right) of the composite-based sleeve, including the composite-based adaptive component, mounted on the forearm of a human subject.

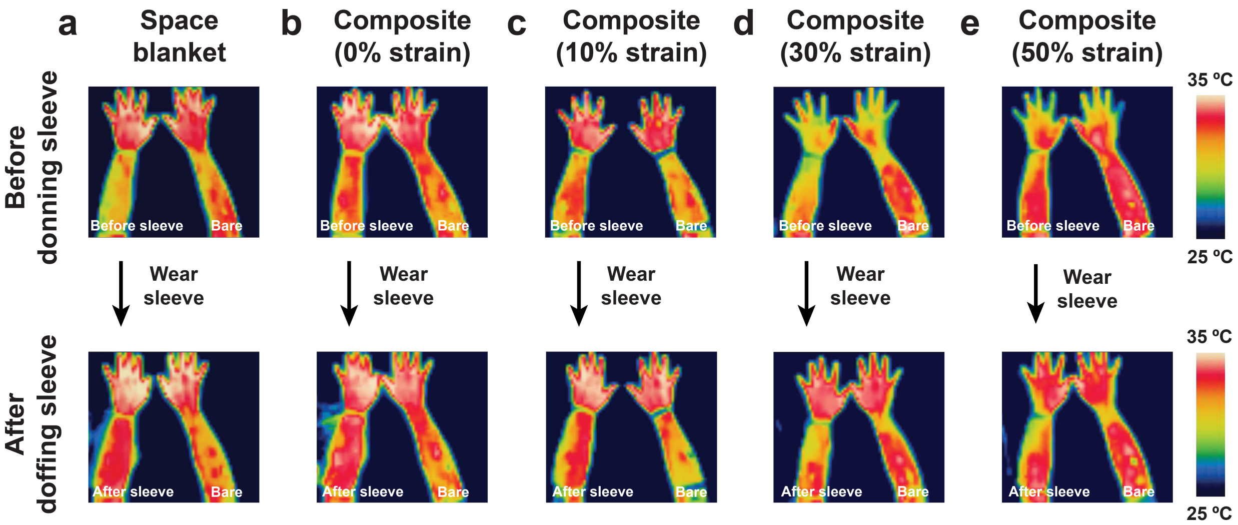

Supplementary Figure 7. Infrared camera images of the local body temperature before and after wearing the mechanically-actuated composite-based sleeve. a, Infrared camera images of an individual's forearms before donning a space blanket-based sleeve on one forearm (top) and the same individual's forearms after doffing the same space blanket-based sleeve (bottom). **b**, Infrared camera images of an individual's forearms before donning a composite-based sleeve, under an applied strain of 0 %, on one forearm (top), and the same individual's forearms after doffing the same composite-based sleeve (bottom). **c**, Infrared camera images of an individual's forearms before donning a composite-based sleeve, under an applied strain of 10 %, on one forearm (top), and the same individual's forearms after doffing the same composite-based sleeve (bottom). **d**, Infrared camera images of an individual's forearms before donning a composite-based sleeve, under an applied strain of 30 %, on one forearm (top), and the same individual's forearms after doffing the same composite-based sleeve (bottom). **e**, Infrared camera images of an individual's forearms before donning a composite-based sleeve, under an applied strain of 50 %, on one forearm (top), and the same individual's forearms after doffing the same composite-based sleeve (bottom).

Supplementary Table 1. Compilation of the key capabilities and figures-of-merit for representative passive and active thermal management systems. The list includes representative examples of the major classes of thermal management systems used in buildings, either in wearable or non-wearable configurations.

Integrated System	Actuation Approach	Thermal Conductivity ($Wm^{-1}K^{-1}$)	Heat Flux (Wm^{-2}) (Temperature Difference)	Power Consumption (Wm^{-2})	Dynamic User Controllability	Reference
Fiberglass batting (made from sand and recycled glass)	Passive	0.037	4 (10 K)*	None	No	*6*
Polyethylene (PE) sheet	Passive	0.041	4.1 (10 K)*	None	No	*6*
Loose-fill cellulose (made from ground-up waste paper)	Passive	0.050	5 (10 K)*	None	No	*6*
Wool garment	Passive	0.031	270 (10 K)*	None	No	*7*
Cotton fabric	Passive	0.035	350 (10 K)*	None	No	*8*
Polyvinyl alcohol fabric	Passive	0.050	500 (10 K)*	None	No	*8*
Space blanket (PET sheet coated with an Al thin film)	Passive	N/A (Emissivity 0.05)	N/A	None	No	*9*
Material made from stacked layers of nanoporous PE, copper, and carbon	Passive	0.025	51 (11.8 or 18.3 K)	Power required for manual flipping	No (dual mode)	*10*
Textile made from stacked layers of nanoporous PE, silver, and cotton	Passive	Not determined/reported	73 (18 K)	None	No	*1*
Silica aerogel	Passive	0.013	13 (10 K)*	None	No	*11*
Carbon aerogel	Passive	0.032	32 (10 K)*	None	No	*12*
Graphene aerogel	Passive	0.005	5 (10 K)*	None	No	*13*
Alumina aerogel	Passive	0.065	65 (10 K)*	None	No	*14*
Woven textile made from boron nitride and polyvinyl alcohol composite fibers	Passive	0.078	780 (10 K)*	None	No	*8*
Woven textile made from porous silk fibroin and chitosan composite fibers	Passive	0.022	Not determined/reported (8 K)	None	No	*15*
Textile made from cotton fibers coated with silver nanowires	Passive	Not determined/reported	Not determined/reported	None	No	*16*
**Composite material made from Cu and SEBS**	**Mechanical**	**0.200**	**260 - 296 (15 K)**	**3 (0.5 s actuation)**	**Yes**	***This work***

Supplementary Table 1 (continued).

Integrated System	Actuation Approach	Thermal Conductivity ($Wm^{-1}K^{-1}$)	Heat Flux (Wm^{-2}) (Temperature Difference)	Power Consumption (Wm^{-2})	Dynamic User Controllability	Reference
Fabric made of graphene fibers	Electrothermal	301.5	Not determined/reported (~350 K†)	49500	Yes	*17*
Cotton fabric knitted with silver filaments	Electrothermal	Not determined/reported	Not determined/reported (30 K)	243†	Yes	*18*
Polyester garment coupled with heating pads made of carbon-filaments	Electrothermal	Not determined/reported	Not determined/reported (32 K)	120†	Yes	*19*
Woven textile made of composite fibers from porous silk fibroin, chitosan, and carbon nanotubes	Electrothermal	0.022	Not determined/reported (12 K)	550†	Yes	*15*
Heat pump device that incorporates a poly(vinylidene fluoride-ter-trifluoroethylene-ter-chlorofluoroethylene) film in an elastocaloric polymer stack	Electrocaloric	Not determined/reported	0-297 (1.4 K‡)	20	Yes	*20*
Heat pump device that incorporates nickel-titanium alloy plates	Elastocaloric	Not determined/reported	Not determined/reported (15.3 K‡)	14398†	Yes	*21*
Heat pump device from a nickel-titanium elastocaloric shape memory alloy	Elastocaloric	Not determined/reported	Not determined/reported (9.4 K‡)	357†	Yes	*22*
Polyester garment coupled with a Bi_2Te_3 alloy thermoelectric cooler/heater and a micro-blower	Thermoelectric	Not determined/reported	116 - 145 (13 K)	Not determined/ reported	Yes	*23*
Mitsubishi HVAC system	HVAC	Not determined/reported	80† (13 K)	19†	Yes	*24*
LG Libero HVAC system	HVAC	Not determined/reported	70† (13 K)	15†	Yes	*25*
**Composite material made from Cu and SEBS**	**Mechanical**	**0.200**	**260 - 296 (15 K)**	**3 (0.5 s actuation)**	**Yes**	***This work***

*The value of the temperature difference and the thickness of the materials were estimated from realistic conditions that might be encountered during use of the relevant integrated system. The heat flux was calculated from Fourier's Law ($q = -k\frac{\Delta T}{\Delta x}$) based on these assumptions. †The value was not explicitly provided but was calculated from the data reported in the associated reference. ‡The temperature difference was generated by the integrated system.

Supplementary Table 2. Compilation of the key capabilities and figures-of-merit for various thermal switches. The list includes representative examples of the major classes of thermal switches that operate via conductive, convective, or radiative mechanisms. Note that the last row summarizes the relevant capabilities and figures-of-merit for cephalopods as the inspiration source.

Materials/Device Configuration	Actuation Method	Dominant Heat Transfer Mechanism	Operating Temperature (K)	Cyclable	Tunable (In Situ)	Soft and Stretchable Form Factor	Area (cm^2)	Response Time	On/Off Ratio (Heat Flux)	On/Off Ratio (Emissivity or Transmittance)	Reference
GeSbTe alloy film	Temperature-induced crystalline-amorphous phase transition	Conduction	Not determined/ reported	No	No	No	1E-4†	< 1 s*	3†	N/A	*26*
GeSbTe alloy film	Temperature-induced crystalline-amorphous phase transition	Radiation	Not determined/ reported	No	No	No	Not determined/ reported	90 s	N/A	10	*27*
W-doped VO_2 nanobeam bridging two suspended micropads	Temperature-induced metal-insulator phase transition	Conduction	Above/below 240 - 340	No	No	No	Not determined/ reported	< 1 s*	1.6†	N/A	*28*
poly(N-isopropylacrylamide) (PNIPAM) aqueous solution	Temperature-induced liquid-solid phase transition	Conduction	Above/below 302	Yes	No	No	4†	Not determined/ reported	2.8†	N/A	*29*
Percolated composite material made of hexadecane/graphite	Temperature-induced liquid-solid phase transition	Conduction	Above/below 291	Yes	No	No	Not determined/ reported	< 1 s*	3.2	N/A	*30*
Ice/water sandwiched between plastic sheets	Temperature-induced liquid-solid phase transition	Conduction	Above/below 273	Yes	No	No	3E-4†	< 1 s*	3.9†	N/A	*31*
Liquid crystal polymer network	Magnetic field-induced alignment of liquid crystals	Conduction	298 - 473	Yes, but hysteresis	No	Yes	0.2	15 min	1.5†	N/A	*32*
Silicone channel filled with a Galinstan liquid droplet and NaOH vapor	Gravity-induced change in thermal contact	Conduction	> 254	Yes	No	No	0.045†	< 1 s*	71	N/A	*33*
Liquid Hg micro-droplet grid on a Si substrate	Mechanically-induced change in thermal contact	Conduction	> 243†	Yes	No	No	0.04	< 1 s*	224	N/A	*34*
Glycerin layer between two Si dies	Electrowetting-induced change in thermal contact	Conduction	272 - 373	Yes	No	No	3.06†	13 s	3†	N/A	*35*
Isopropanol droplet on a Teflon-covered Al substrate	Electrically-controlled Leidenfrost effect	Convection	> 413	No	Yes	No	0.0063	< 1 s*	20	N/A	*36*
**Composite material made from Cu and SEBS**	**Mechanically-induced change in infrared properties**	**Radiation**	**233 - 373**	**Yes**	**Yes**	**Yes**	**170**	**< 1 s**	**1.2**	**> 25**	***This work***

Supplementary Table 2 (continued).

Materials/Device Configuration	Actuation Method	Dominant Heat Transfer Mechanism	Operating Temperature (K)	Cyclable	Tunable (In Situ)	Soft and Stretchable Form Factor	Area (cm^2)	Response Time	On/Off Ratio (Heat Flux)	On/Off Ratio (Emissivity or Transmittance)	Reference
Cu/Co multilayer film on a MgO substrate	Magnetic field-induced alignment of electron spin	Conduction	Not determined/ reported	Yes	Yes	No	2.8E-7†	< 1 s*	1.8†	N/A	*37*
Polycarbonate thermal valve filled with magnetite nanofluids	Non-uniform magnetic field-controlled convection modulation	Convection	Not determined/ reported	Yes	Yes	No	4.9†	Not determined/ reported	13	N/A	*38*
Two concentric Cu cylinders filled with He gas	Pressure-controlled change in gas thermal conductivity	Conduction	90 - 291.2	Yes	Yes	No	7.9†	Not determined/ reported	512	N/A	*39*
Polyurethane lamellar foam filled with He gas	Pressure-controlled change in gas thermal conductivity	Conduction	273 - 423	Yes	Yes	Yes	2†	Not determined/ reported	10.5†	N/A	*40*
Heat pipe filled with water and N_2 gas	Temperature gradient-induced evaporation/condensation of working fluid	Conduction	Not determined/ reported	Yes	Yes	No	70†	Not determined/ reported	200†	N/A	*41*
Proton-conducting polymer-gel electrolytes sandwiched between gold and ITO electrodes	Electrochemical doping-induced modulation of the infrared reflectance	Radiation	Not determined/ reported	Yes	Yes	No	4.9†	9 s	N/A	2.3†	*42*
$WO_3/Ta_2O_5/WO_3$ film sandwiched between aluminum electrodes	Electrochemical doping-induced infrared emittance modulation	Radiation	Not determined/ reported	Yes	Yes	No	Not determined/ reported	15 min†	N/A	1.5†	*43*
Polyaniline film sandwiched between polymeric electrodes	Electrochemical doping-induced modulation of mid-infrared optical properties	Radiation	Not determined/ reported	Yes	Yes	No	Not determined/ reported	Not determined/ reported	N/A	1.7†	*44*
$WO_3 \cdot 2H_2O$ nanosheets sandwiched between flexible electrodes	Electric field-induced change in optical properties	Radiation	Not determined/ reported	Yes	Yes	Yes	2.25†	10 s	N/A	~ 3.3†	*45*
**Composite material made from Cu and SEBS**	**Mechanically-induced change in infrared properties**	**Radiation**	**233 - 373**	**Yes**	**Yes**	**Yes**	**170**	**< 1 s**	**1.2**	**> 25**	***This work***
Cephalopods (e.g. the *Loligo pealeii* squid)	Neurally-activated morphological and areal changes	N/A	277 - 303	Yes	Yes	Yes	~300‡	Few seconds (whole animal), < 1s (chromatophore)	N/A	N/A	*46-60*

*The value was estimated from the operating mechanism of the device. †The value was not explicitly provided but was calculated from the data reported in the associated reference. ‡The value can vary significantly but was roughly estimated from commercially-purchased squid specimens.

Supplementary Table 3. Compilation of parameters used to model heat transfer. The list includes various types of commercial cloth/fabrics and the composite material at various strains described in the manuscript.

**Fabric/ Material**	**Reflectance On Inner Side, ρ_i** *	**Reflectance On Outer Side, ρ_o** *	**Transmittance, τ_c** *	**Thermal Conductivity, k_c ($Wm^{-1}K^{-1}$)** †	**Thickness, t_c (mm)**	**Calculated Setpoint Temperature, T_e (°C)**
Space Blanket	0.99	0.53	0.01	0.15 [61]	0.02‡	14.3
Omniheat	0.41	0.21	0.01	0.04 [62]	1.85‡	18.9
Wool	0.10	0.10	0.01	0.04 [63]	2.20‡	20.5
Acrylic	0.23	0.23	0.04	0.036 [7]	1.25‡	20.7
Flannel	0.14	0.14	0.01	0.045-0.055 [64,65]	0.65‡	21.9
Polyester	0.17	0.17	0.03	0.04 [62]	0.37‡	22.0
Silk	0.14	0.14	0.05	0.082 [66,67]	0.13‡	22.7
Linen	0.13	0.13	0.04	0.12 [66,67]	0.30‡	22.7
Cotton	0.13	0.13	0.08	0.045-0.055 [64,65]	0.24‡	22.8
Rayon	0.12	0.12	0.07	0.038-0.048 [64]	0.10‡	23.0
Composite (0% Strain)	0.96	0.64	0.01	0.19 [68]	0.030‡	14.5
Composite (10% Strain)	0.88	0.60	0.05	0.19 [68]	0.029⁋	16.3
Composite (30% Strain)	0.73	0.51	0.15	0.19 [68]	0.026⁋	19.2
Composite (40% Strain)	0.68	0.48	0.20	0.19 [68]	0.025⁋	20.2
Composite (50% Strain)	0.63	0.46	0.24	0.19 [68]	0.025⁋	20.9
Composite (70% Strain)	0.58	0.43	0.30	0.19 [68]	0.023⁋	21.7
Composite (100% Strain)	0.51	0.40	0.38	0.19 [68]	0.021⁋	22.7

*The reflectance and transmittance were normalized with respect to the highly reflective space blanket. †A range of thermal conductivities has been reported for many textiles and fabrics[69]. ‡The thickness was measured experimentally. ⁋The thickness was calculated from measurements by using Poisson's ratio of 0.5 for a rubber.

Supplementary References

1. Cai, L. et al. Warming up human body by nanoporous metallized polyethylene textile. *Nat. Commun.* **8**, 496 (2017).

2. Tong, J. K. et al. Infrared-transparent visible-opaque fabrics for wearable personal thermal management. *ACS Photonics* **2**, 769-778 (2015).

3. Hanssen, L. M. & Snail, K. A. Integrating spheres for mid- and near-infrared reflection spectroscopy. In *Handbook of Vibrational Spectroscopy.* (Chalmers, J. M. & Griffiths, P. R., eds.) (John Wiley & Sons Ltd, Chichester, 2002).

4. Gindele, K., Köhl, M. & Mast, M. Spectral reflectance measurements using an integrating sphere in the infrared. *Appl. Opt.* **24**, 1757-1760 (1985).

5. Pike Technologies, Inc. Reference Standards – For Calibrating FTIR Spectrometers. https://www.piketech.com/files/pdfs/PIKE_Standards_Data_Sheet.pdf.

6. Al-Homoud, M. S. Performance characteristics and practical applications of common building thermal insulation materials. *Build. Environ.* **40**, 353-366 (2005).

7. Hashan, M. M. et al. Functional properties improvement of sock items using different types of yarn. *Inter. J. Text. Sci.* **6**, 34-42 (2017).

8. Gao, T. et al. Three-dimensional printed thermal regulation textiles. *ACS Nano* **11**, 11513-11520 (2017).

9. Finckenor, M. M. & Dooling, D. “Multilayer Insulation Material Guidelines” (NASA/TP-1999-209263, 1999).

10. Hsu, P.-C. et al. A dual-mode textile for human body radiative heating and cooling. *Sci. Adv.* **3**, e1700895 (2017).

11. Baetens, R., Jelle, B. P. & Gustavsen, A. Aerogel insulation for building applications: a state-of-the-art review. *Energ. Buildings* **43**, 761-769 (2011).

12. Jia, X. et al. Strong and machinable carbon aerogel monoliths with low thermal conductivity prepared via ambient pressure drying. *Carbon* **108**, 551-560 (2016).

13. Xie, Y. et al. Interface-mediated extremely low thermal conductivity of graphene aerogel. *Carbon* **98**, 381-390 (2016).

14. Zu, G. et al. Preparation and characterization of monolithic alumina aerogels. *J. Non-Cryst. Solids* **357**, 2903-2906 (2011).

15. Cui, Y., Gong, H., Wang, Y., Li, D. & Bai, H. A thermally insulating textile inspired by polar bear hair. *Adv. Mater.* **30**, 1706807 (2018).

16. Hsu, P.-C. et al. Personal thermal management by metallic nanowire-coated textile. *Nano Lett.* **15**, 365-371 (2015).

17. Li, Z., Xu, Z., Liu, Y., Wang, R. & Gao, C. Multifunctional non-woven fabrics of interfused graphene fibres. *Nat. Commun.* **7**, 13684 (2016).

18. Hao, L. et al. Development and characterization of flexible heating fabric based on conductive filaments. *Measurement* **45**, 1855-1865 (2012).

19. Song, W., Lai, D. & Wang, F. Evaluating the cold protective performance (CPP) of an electrically heated garment (EHG) and a chemically heated garment (CHG) in cold environments. *Fibers Polym.* **16**, 2689-2697 (2015).

20. Ma, R. et al. Highly efficient electrocaloric cooling with electrostatic actuation. *Science* **357**, 1130-1134 (2017).

21. Tušek, J. et al. A regenerative elastocaloric heat pump. *Nat. Energy* **1**, 16134 (2016).

22. Ossmer, H. et al. Energy-efficient miniature-scale heat pumping based on shape memory alloys. *Smart Mater. Struct.* **25**, 085037 (2016).

23. Zhao, D. et al. Personal thermal management using portable thermoelectrics for potential building energy saving. *Appl. Energy* **218**, 282-291 (2018).

24. Mitsubishi Electric, "M-Series High Performance Single Zone Systems" (FHBRO_06.14 15K OA, www.mitsubishicomfort.com/sites/default/files/manual/fh-series_highrise_brochure.pdf).

25. LG Electronics, "LA120HYV1 Art Cool Premier Single Zone Inverter" (SB-SingleZone Premier-LA120HYV1-02-15, LG, www.refripartesrd.com/uploads/LAN120HYV1,%20LAU120HYV1.pdf).

26. Lee, J. et al. Phonon and electron transport through $Ge_2Sb_2Te_5$ films and interfaces bounded by metals. *Appl. Phys. Lett.* **102**, 191911 (2013).

27. Du, K.-K. et al. Control over emissivity of zero-static-power thermal emitters based on phase-changing material GST. *Light Sci. Appl.* **6**, e16194 (2017).

28. Lee, S. et al. Anomalously low electronic thermal conductivity in metallic vanadium dioxide. *Science* **355**, 371–374 (2017).

29. Cottril, A. L., Wang, S., Liu, A. T., Wang, W.-J. & Strano, M. S. Dual phase change thermal diodes for enhanced rectification ratios: theory and experiment. *Adv. Energy Mater.* **8**, 1702692 (2018).

30. Zheng, R., Gao, J., Wang, J. & Chen, G. Reversible temperature regulation of electrical and thermal conductivity using liquid-solid phase transitions. *Nat. Commun.* **2**, 289 (2011).

31. Lubner, S. D. et al. Reusable bi-directional 3ω sensor to measure thermal conductivity of 100-μm thick biological tissues. *Rev. Sci. Instrum.* **86**, 014905 (2015).

32. Shin, J. et al. Thermally functional liquid crystal networks by magnetic field driven molecular orientation. *ACS Macro Lett.* **5**, 955-960 (2016).

33. Yang, T. et al. Millimeter-scale liquid metal droplet thermal switch. *Appl. Phys. Lett.* **112**, 063505 (2018).

34. Cho, J., Richards, C., Bahr, D., Jiao, J. & Richards, R. Evaluation of contacts for a MEMS thermal switch. *J. Micromech. Microeng.* **18**, 105012 (2008).

35. McLanahan, A. R., Richards, C. D. & Richards, R. F. A dielectric liquid contact thermal switch with electrowetting actuation. *J. Micromech. Microeng.* **21**, 104009 (2011).

36. Shahriari, A., Wurz, W. & Bahadur, V. Heat transfer enhancement accompanying Leidenfrost state suppression at ultrahigh temperatures. *Langmuir* **30**, 12074-12081 (2014).

37. Kimling, J. et al. Spin-dependent thermal transport perpendicular to the planes of Co/Cu multilayers. *Phys. Rev. B* **91**, 144405 (2015).

38. Seshadri, I. et al. Gating heat transport by manipulating convection in a magnetic nanofluid. *Appl. Phys. Lett.* **102**, 203111 (2013).

39. Bywaters, R. P. & Griffin, R. A. A gas-gap thermal switch for cryogenic applications. *Cryogenics* **13**, 344-349 (1973).

40. Chau, M. et al. Reversible transition between isotropic and anisotropic thermal transport in elastic polyurethane foams. *Mater. Horiz.* **4**, 236-241 (2017).

41. Leriche, M., Harmand, S., Lippert, M. & Desmet, B. An experimental and analytical study of a variable conductance heat pipe: application to vehicle thermal management. *Appl. Therm. Eng.* **38**, 48-57 (2012).

42. Topart, P. & Hourquebie, P. Infrared switching electroemissive devices based on highly conducting polymers. *Thin Solid Films* **352**, 243-248 (1999).

43. Franke, E. B., Trimble, C. L., Schubert, M., Woollam, J. A. & Hale, J. S. All-solid-state electrochromic reflectance device for emittance modulation in the far-infrared spectral region. *Appl. Phys. Lett.* **77**, 930-932 (2000).

44. Li, H., Xie, K., Pan, Y., Yao, M. & Xin, C. Variable emissivity infrared electrochromic device based on polyaniline conducting polymer. *Synth. Met.* **159**, 1386-1388 (2009).

45. Liang, L. et al. High-performance flexible electrochromic device based on facile semiconductor-to-metal transition realized by $WO_3 \cdot 2H_2O$ ultrathin nanosheets. *Sci. Rep.* **3**, 1936 (2013).

46. Mäthger, L. M., Denton, E. J., Marshall, N. J. & Hanlon, R. T. Mechanisms and behavioural functions of structural coloration in cephalopods. *J. R. Soc. Interface* **6** (suppl. 2), S149-S163 (2009).

47. Hanlon, R. Cephalopod dynamic camouflage. *Curr. Biol.* **17**, R400-R404 (2007).

48. Phan, L. et al. Dynamic materials inspired by cephalopods. *Chem. Mater.* **28**, 6804-6816 (2016).

49. Mäthger, L. M. & Hanlon, R. T. Malleable skin coloration in cephalopods: selective reflectance, transmission and absorbance of light by chromatophores and iridophores. *Cell Tissue Res.* **329**, 179-186 (2007).

50. Wardill, T. J., Gonzalez-Bellido, P. T., Crook, R. J. & Hanlon, R. T. Neural control of tuneable skin iridescence in squid. *Proc. Biol. Sci.* **279**, 4243-4252 (2012).

51. Deravi, L. F. et al. The structure-function relationships of a natural nanoscale photonic device in cuttlefish chromatophores. *J. R. Soc. Interface* **11**, 20130942 (2014).

52. DeMartini, D. G., Krogstad, D. V. & Morse, D. E. Membrane invaginations facilitate reversible water flux driving tunable iridescence in a dynamic biophotonic system. *Proc. Natl. Acad. Sci. U.S.A.* **110**, 2552-2556 (2013).

53. Messenger, J. B. Cephalopod chromatophores: neurobiology and natural history. *Biol. Rev. Camb. Philos. Soc.* **76**, 473-528 (2001).

54. Wood, J. B. & O'Dor, R. K. Do larger cephalopods live longer? Effects of temperature and phylogeny on interspecific comparisons of age and size at maturity. *Mar. Biol.* **136**, 91-99 (2000).

55. Kreit, E. et al. Biological versus electronic adaptive coloration: How can one inform the other? *J. R. Soc. Interface* **10**, 20120601 (2013).

56. Vidal, E. "*Advances in Cephalopod Science: Biology, Ecology, Cultivation and Fisheries*" vol. 67 of *Advances in Marine Biology* (Elsevier, 2014).

57. Ramirez, M. D. & Oakley, T. H. Eye-independent, light-activated chromatophore expansion (LACE) and expression of phototransduction genes in the skin of Octopus bimaculoides. *J. Exp. Biol.* **218**, 1513-1520 (2015).

58. Kirkwood, K. M., Bell, G., Kuzirian, A. M., Hanlon, R. T. & Wetzel, E. D. Mechanics of the cephalopod chromatophore layer: structural characterization of cephalopod chromatophores. *Proc. ASME* **53073**, 427-428 (2011).

59. Iglesias, J., Fuentes, L. & Villanueva, R. *Cephalopod Culture* (Springer, 2014).

60. O'dor, R. K., Mangold, K., Boucher-Rodoni, R., Wells, M. J. & Wells, J. Nutrient absorption, storage and remobilization in octopus vulgaris. *Mar. Behav. Physiol.* **11**, 239-258 (1984).

61. Meseguer, J., Pérez-Grande, I. & Sanz-Andrés, A. *Spacecraft Thermal Control* (Woodhead Publishing, 2012).

62. Ehrmann, A. & Blachowicz T. Thermal Properties of Textiles. In *Examination of Textiles Mathematical and Physical Methods*, 113-123 (Springer, 2017).

63. Young, H. D. *University Physics*, 7th edition (Addison Wesley, 1992).

64. Majumdar, A., Mukhopadhyay, S. & Yadav, R. Thermal properties of knitted fabrics made from cotton and regenerated bamboo cellulosic fibres. *Int. J. Therm. Sci.* **49**, 2042-2048 (2010).

65. Prakash, C. & Ramakrishnan, G. Study of thermal properties of bamboo/cotton blended single jersey knitted fabrics. *Arab. J. Sci. Eng.* **39**, 2289-2294 (2014).

66. Kawabata, S. & Rengasamy, R. S. Thermal conductivity of unidirectional fibre composites made from yarns and computation of thermal conductivity of yarns. *Indian J. Fibre Text.* **27**, 217-223 (2002).

67. Thermtest. Thermal conductivity testing of fabrics. https://thermtest.com/thermal-conductivity-testing-of-fabrics.

68. Tsutsumi, N. Thermal conductivity of multiphase polymers. In *Structure and Properties of Multiphase Polymeric Materials*, (eds Araki, T., Shibayama, M. & Tran-Cong, Q.) Chapter 11, 393-422 (Marcel-Dekker, Inc., 1998).

69. Salopek Čubríc, I., Skenderi, Z., Mihelić-Bogdanić, A. & Andrassy, M. Experimental study of thermal resistance of knitted fabrics. *Exp. Therm. Fluid Sci.* **38**, 223-228 (2012).
