# Supplementary material for: A dynamic thermoregulatory material inspired by squid skin
Source: Nat Commun. 2019 Apr 29;10:1947. doi: 10.1038/s41467-019-09589-w (PMC6488639; doi:10.1038/s41467-019-09589-w)
Supplement: Supplementary file 3 — Description of Additional Supplementary Files [file 41467_2019_9589_MOESM3_ESM.docx]

**Title:** Supplementary Movie 1
**Description:** An infrared camera video of the mechanical actuation of a composite-based directly sleeve on the forearm of a human subject, demonstrating the real time modulation of the local heat flux to the surrounding environment.
